# Dysregulation in Multiple Transcriptomic Endometrial Pathways Is Associated with Recurrent Implantation Failure and Recurrent Early Pregnancy Loss

**DOI:** 10.3390/ijms232416051

**Published:** 2022-12-16

**Authors:** Norhayati Liaqat Ali Khan, Tamer Nafee, Tingting Shao, Amber Rose Hart, Sarah Elliott, Bolarinde Ola, Paul Roy Heath, Alireza Fazeli

**Affiliations:** 1 Academic Unit of Reproductive and Developmental Medicine, Department of Oncology and Metabolism, The Medical School, University of Sheffield, Sheffield S10 2HQ, UK; 2 Faculty of Dentistry, Universiti Teknologi MARA (UiTM), Sungai Buloh 47000, Malaysia; 3 Sheffield Teaching Hospitals NHS Foundation Trust, University of Sheffield, Sheffield S10 2HQ, UK; 4 Sheffield Institute for Translational Neuroscience (SITRAN), University of Sheffield, Sheffield S10 2HQ, UK; 5 Department of Pathophysiology, Institute of Biomedicine and Translational Medicine, Faculty of Medicine, University of Tartu, 14B Ravila, 50411 Tartu, Estonia; 6 Institute of Veterinary Medicine and Animal Sciences, Estonian University of Life Sciences, 51014 Tartu, Estonia

**Keywords:** recurrent implantation failure (RIF), recurrent early pregnancy loss (REPL), differentially expressed genes (DEGs), gene expression profile, endometrial biopsy, endometrial pathways

## Abstract

Overlapping disease aetiologies associated with multiple altered biological processes have been identified that change the endometrial function leading to recurrent implantation failure (RIF) and recurrent early pregnancy loss (REPL). We aimed to provide a detailed insight into the nature of the biological malfunction and related pathways of differentially expressed genes in RIF and REPL. Endometrial biopsies were obtained from 9 women experiencing RIF, REPL and control groups. Affymetrix microarray analysis was performed to measure the gene expression level of the endometrial biopsies. Unsupervised clustering of endometrial samples shows scattered distribution of gene expression between the RIF, REPL and control groups. 2556 and 1174 genes (*p* value < 0.05, Fold change > 1.2) were significantly altered in the endometria of RIF and REPL patients’ group, respectively compared to the control group. Downregulation in Kyoto Encyclopedia of Genes and Genomes (KEGG) pathways of the differentially expressed genes (DEGs) in RIF and REPL including ribosome and oxidative phosphorylation pathways. Gene Ontology (GO) analysis revealed ribosomes and mitochondria inner membrane as the most significantly downregulated cellular component (CC) affected in RIF and REPL. Determination of the dysregulated genes and related biological pathways in RIF and REPL will be key in understanding their molecular pathology and of major importance in addressing diagnosis, prognosis, and treatment issues

## 1. Introduction

Parenthood and starting a family largely are wanted and anticipated life goals for most people. However, upon starting this journey to parenthood, numerous couples find their plans interrupted and complicated by infertility. Infertility, defined as inability to conceive after having frequent and unprotected sex for 12 months [1], has been shown statistically to affect 1 out of 4 couples [2] in developing countries and globally it affects about 48.5 million couples [3]. Consequently, couples and women struggling with infertility experience great emotional distress, feelings of grief as well as potential stigmatization and relationship strain [4,5]. For these couples many seek the use of assisted reproductive technologies (ART). Despite significant advances in such technologies over the decades, the outcome of ART is still largely unsuccessful. A key constraining phase in successful ART is implantation failure of seemingly competent embryos, causing a reported success rate of less than 30% of high-quality embryo transferred being successfully implanted [6]. 

A large proportion of couples undergoing ART experience this failure multiple times, referred to as recurrent implantation failure (RIF), an upsetting occurrence that can add to the emotional and financial stress of couples struggling to achieve a pregnancy. RIF is defined as failure to achieve clinical pregnancy after three or more transfers of high-quality embryos in a minimum of three fresh or frozen cycles of in vitro fertilization (IVF) recognized by the presence of an intrauterine gestational sac during ultrasonography [7]. Multiple failed cycles can leave couples frustrated and desperate for explanations as to the cause of this failure. Numerous aetiologies for this implantation failure have been suggested, such as uterine and embryo related factors as well as maternal age and other multifactorial effectors including sperm quality and chromosomal anomalies [8]. However, in most instances of implantation failure no well-defined root cause is identifiable. 

Another distinctly different challenge to reproductive medicine and fertility is recurrent early pregnancy loss (REPL), defined as two or more consecutive pregnancy losses before 20 weeks of gestation [9]. Pregnancy loss affects around 1–5% of women of child-bearing age and occurs in approximately 12–15% of clinically recognized normal pregnancies without any medical assistance [10]. This condition also occurs in 10% of patients undergoing ART within the 30% of the successful rate of IVF procedures. Like RIF, several aetiologies have been associated with REPL including genetic factors, anatomical factors, autoimmune reactions, infections and endocrine factors [11,12,13]. However, although REPL is a distinctly different disorder, there are similarities and many overlapping risk factors and causes with RIF suggesting possible genetic, transcriptomics and/or proteomics link between the two [14].

Of these overlapping factors, multiple altered biologic processes in the endometrium have been identified which could change the endometrium’s functioning ultimately leading to RIF and/or REPL. Some of these alterations are due to the aberrant expression of several different genes and proteins which have been identified following the introduction of high-throughput transcriptome analysis technologies, transcriptomic studies utilizing microarray analysis or RNA sequencing (RNA-Seq) [15]. For example, in a study by Bastu et al. (2019), through analysis of differentially expressed genes (DEGs) of RIF patients, various pathways such as circadian rhythm, pathways in cancer, citrate cycle, the immune system and inflammation, and others were identified [16].

In a further study of REPL; immunity, angiogenesis, apoptosis, cell signaling related pathways and many more were also reported to be altered [17]. Several studies have focused on various aspects of the disease or other accompanying conditions such as, polycystic ovary syndrome [18] prior to and in the window of implantation [19], in recurrent miscarriages [20] and with various hormone treatments [21]. However, few earlier studies have analyzed and compared the endometrial RNA in RIF and REPL and so such conditions continue to be not fully understood, with an efficient diagnosis and or treatment remaining elusive. Hence, in this study, we aimed to provide a detailed view of the biological malfunction and related pathways of DEGs in RIF and REPL. Determination of their aetiologies and identification of dependable biomarkers will be key in understanding their molecular pathology and of major importance in addressing diagnosis, prognosis, and treatment issues. We conclude that, determination of the dysregulated genes and its related biological pathways in RIF and REPL will be key in understanding their molecular pathology and of major importance in addressing diagnosis, prognosis, and treatment issues.

## 2. Results

In the present study, Pipelle endometrial biopsy samples were collected during the midluteal phase of menstrual cycle (7–10 days prior to menstruation) from RIF, REPL and control group patients attending fertility clinic at Jessop Wing aged 40 years old and below following the set inclusion and exclusion criteria (Table 1).

### 2.1. Midluteal Phase Endometrial Tissue for RIF, REPL and Control Group Exhibited Distinct mRNA Expression Profiles

The transcription profiles of endometrial cells from Pipelle endometrial biopsy of RIF patients (*n* = 3), REPL patients (*n* = 3) and control group (*n* = 3) during the midluteal phase of the menstrual cycle were generated using Affymetrix GeneChip^®^ Human Clariom S Array (Affymetrix, Sunnyvale, CA, USA). To identify DEGs in RIF and REPL group of patients versus control group, comparisons were made between the transcription profiles of endometrial cells obtained from RIF and REPL group of patients and the control group, respectively.

A total number of 2556 unique genes were differentially expressed between RIF patients and control group of individuals (1192 upregulated and 1364 downregulated) and 1172 unique genes were differentially expressed between REPL and control group of individuals (543 upregulated and 629 downregulated), respectively. In total, 540 of DEGs in RIF and REPL patients overlapped.

### 2.2. Unsupervised Clustering of Microarray Data Sets of the Endometrial Biopsies

**Figure 1:**
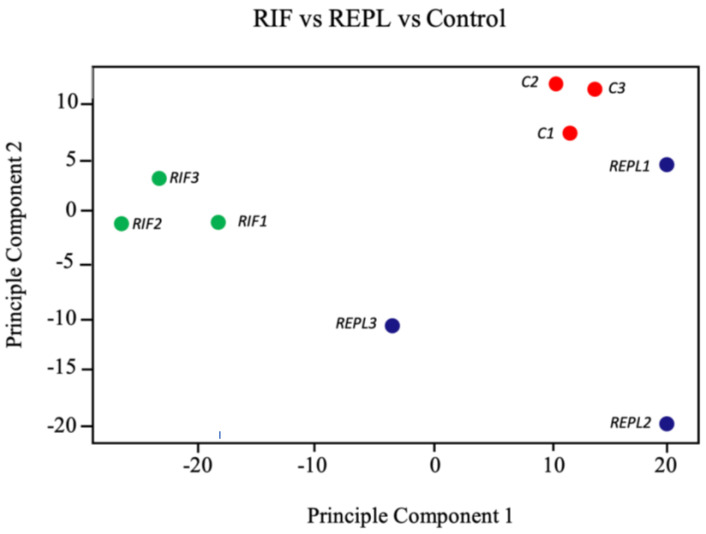
Unsupervised clustering of endometrial samples biopsied in midluteal phase of unfiltered data showing scattered distribution of the participants gene expression. Multidimensional scaling plots were generated in Python 3.8.3 for differentially expressed genes (DEGs) with *p* value < 0.05 and fold change >1.2 or <−1.2 generated. Sample groups: C (red): control group; RIF (green): patients with recurrent implantation failure; REPL (blue): patients with recurrent early pregnancy loss.

To explore the microarray data in an unsupervised manner, different multiple dimension scaling (MDS) plots using Python 3.8.3 were generated based on DEGs (*p* < 0.05; fold change >1.2 or <−1.2) generated after comparing RIF with REPL (Figure 1) between each pair of Affymetrix microarray samples using Transcription Analysis Console (TAC).

A complete segregation of samples derived from RIF, REPL and control group was observed. This data suggest that an mRNA expression signature could potentially exist in RIF and REPL with the right set of genes. 

### 2.3. Top 20 DEGs in Endometrial Tissue of RIF and REPL Group of Patients

**Figure 2:**
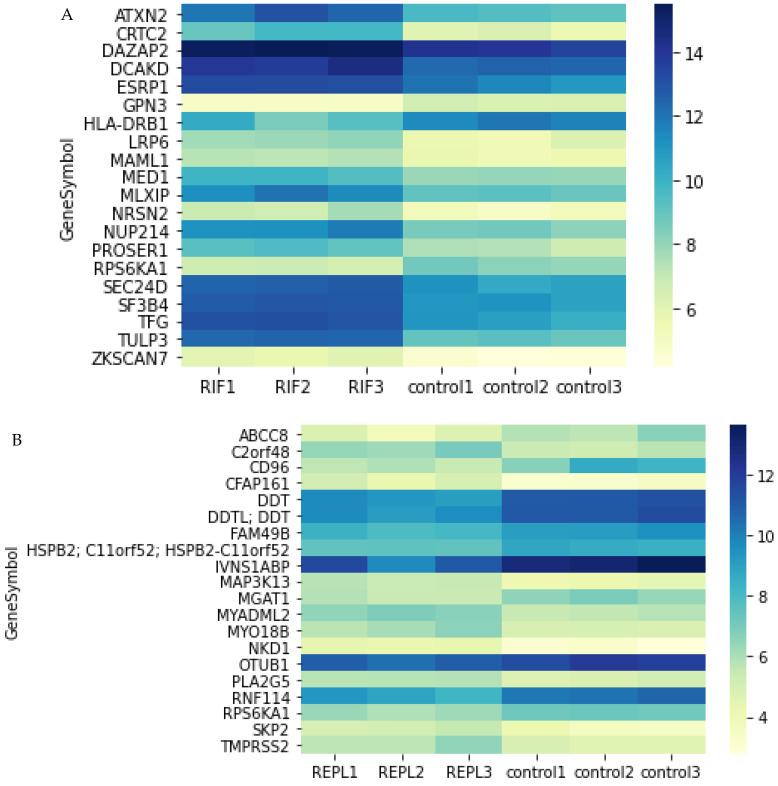
Heatmaps showing a score evaluated based on the expression level for the top 20 genes with lowest *p*-value (yellow =  lowest score, blue =  score of 7 or higher). Each column represents each participant (**A**) RIF (**B**) REPL.

The top 20 DEGs obtained from RIF and REPL patient groups were selected to illustrate the very specific regulation of gene expression in endometrium at midluteal phase (Figure 2). The gene symbol, gene name and the fold change of the top 20 DEGs in RIF and REPL group of patients are illustrated (Table 2 and Table 3).

### 2.4. Identification of Biological Pathways

#### 2.4.1. KEGG Pathway

In the present study, with Database for Annotation, Visualization and Integrated Discovery (DAVID), Kyoto Encyclopedia of Genes and Genomes (KEGG) pathway enrichment analysis representing the knowledge on high-level functions of the biological system was conducted. 42 significant pathways (*p* < 0.05) that correlated to the DEGs in RIF group of patients were identified including 17 upregulated and 25 downregulated KEGG pathways. From these 17 significantly upregulated KEGG pathways, overall, less than 21% of the genes were affected, in each of the pathways. Only 11.6% of the genes were upregulated, thus this observation gave us an idea of an increase in the RNA translation activity in RIF patients.

**Figure 3:**
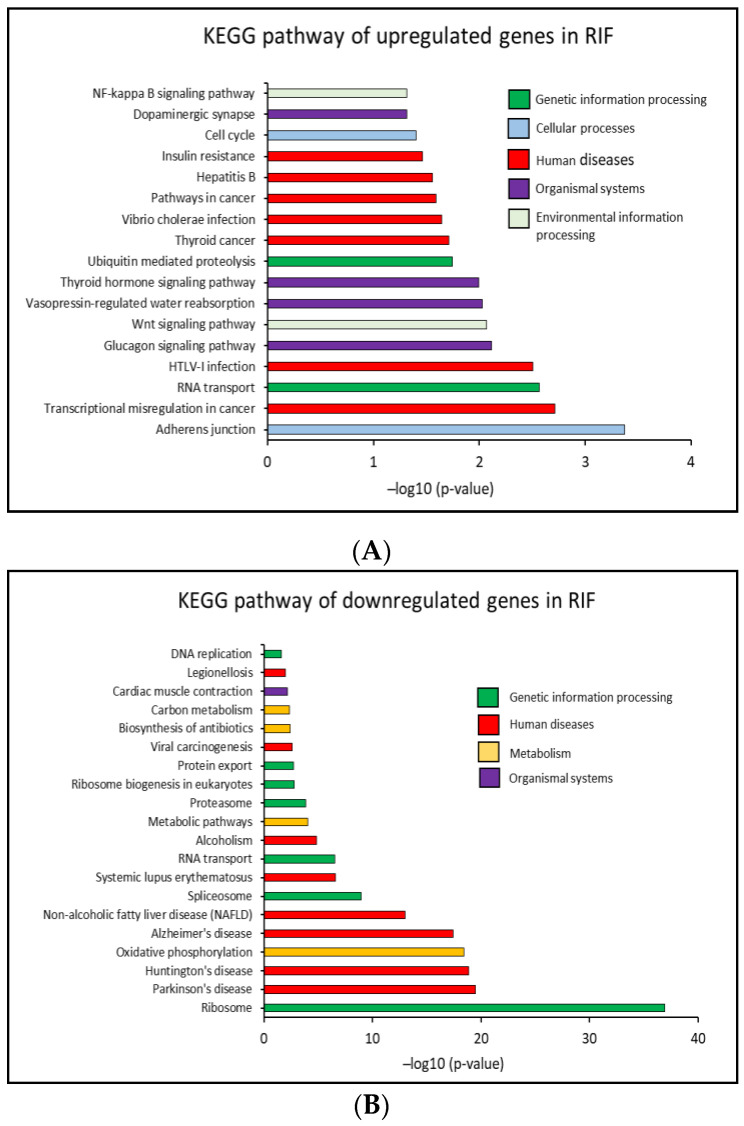
Bioinformatics analysis of DEGs in RIF groups patients compared to control groups. Top 20 (**A**) upregulated and (**B**) downregulated KEGG pathways annotation with significant *p*-value < 0.05. in RIF.

In addition, from the 25 significantly downregulated KEGG pathways in RIF patients, 50% and 20.9% of the genes in ribosome and RNA transport pathway were affected, respectively. Again, these two pathways are closely related to RNA translation to protein and may have a significant function in cell biology. Surprisingly, the genes that were altered in the RNA transport pathway in RIF patients were totally different from each other. Moreover, transcriptomes involved in the oxidative phosphorylation pathway which is involved in cellular ATP production in the mitochondria were also affected in RIF patients with 36.1% of the genes downregulated. This may point to the disruption of energy metabolism in the affected system. The top 20 significantly upregulated and downregulated KEGG pathways in RIF patients are shown in Figure 3A,B. The KEGG pathways, affected genes percentage and the genes symbol are shown in Supplementary Tables S1 and S2.

In contrast, in REPL patients 22 significant altered KEGG pathways (*p* < 0.05) were identified, including 3 pathways which were significantly upregulated and 19 pathways that were downregulated compared to the control group. Pathways upregulated were mostly associated with metabolic activities and this impacted less than 12% of the genes in each of the pathway. Furthermore, the pathways that were downregulated in REPL patients were more diverse and the affected genes were less than 23%, overall. Cellular ATP production in the mitochondria seems to be affected with 11.3% of the genes downregulated in the oxidative phosphorylation pathway. 13.2% of the genes were downregulated in the ribosome pathway indicating disturbances in translation activity.

**Figure 4:**
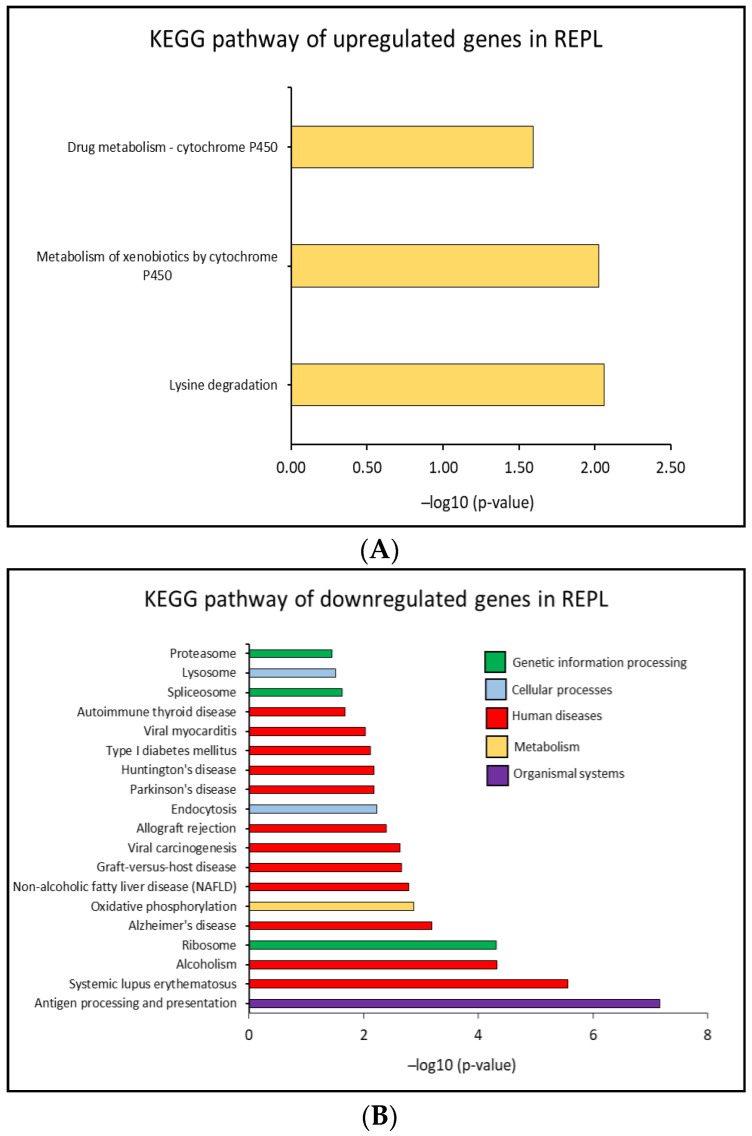
Bioinformatics analysis of DEGs in REPL groups patients compared to control group. Top 20 (**A**) upregulated and (**B**) downregulated KEGG pathways annotation with significant *p*-value < 0.05 in REPL.

The major affected pathways were similar to those found in the RIF patient analysis. Furthermore, 8.3% and 9.1% of the genes related to endocytosis and lysosome pathways, respectively, were also downregulated. These two pathways are closely related to extracellular vesicles biogenesis, secretion, and uptake. The top 20 significantly upregulated and downregulated KEGG pathways in REPL patients are shown in Figure 4A,B. The KEGG pathways, affected genes percentage and the genes symbol are shown in Supplementary Tables S3 and S4. 

#### 2.4.2. GO Analysis

We investigated GO analysis of DEGs in RIF and REPL group of patients from control group output using DAVID. This analysis was crucial to gain insight into the molecular regulatory mechanisms of RIF and REPL. The findings highlighted three main domains of the ontology Molecular function (GO MF), Cellular component (GO CC) and Biological process (GO BP) and its association with DEGs that were significantly enriched (*p* < 0.05).

In RIF participants, GO MF of the DEGs revealed a total of 106 related functions (52 upregulated, 54 downregulated). In addition, for GO CC, 120 cellular regions were significantly affected (39 upregulated, 81 downregulated). Interestingly, GO BP alteration in the gene expression, with a total of 241 altered processes (92 upregulated, 149 downregulated) suggests multiple effects underlying molecular activity in RIF.

**Figure 5:**
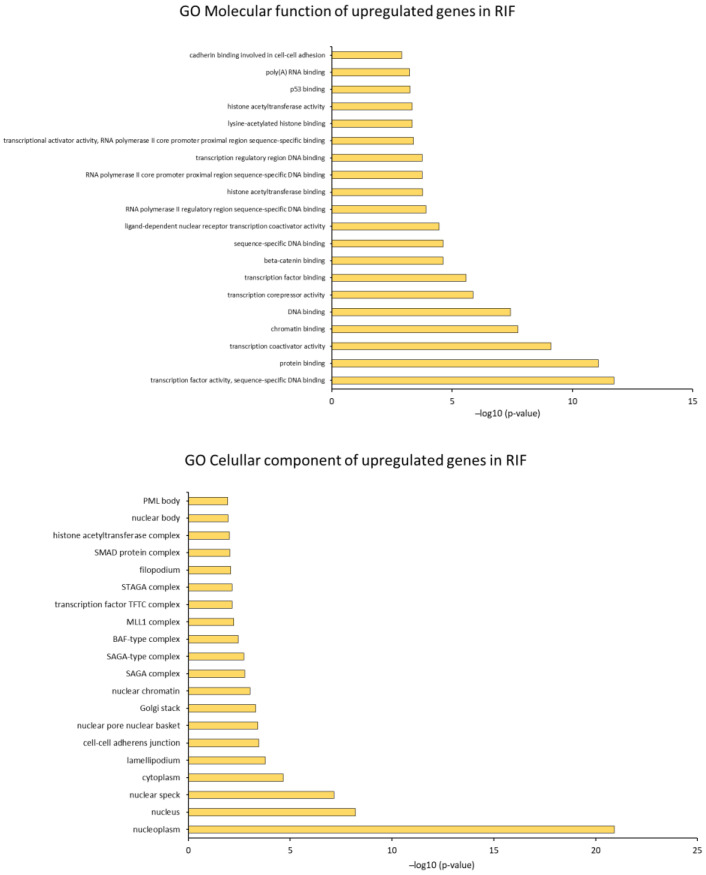

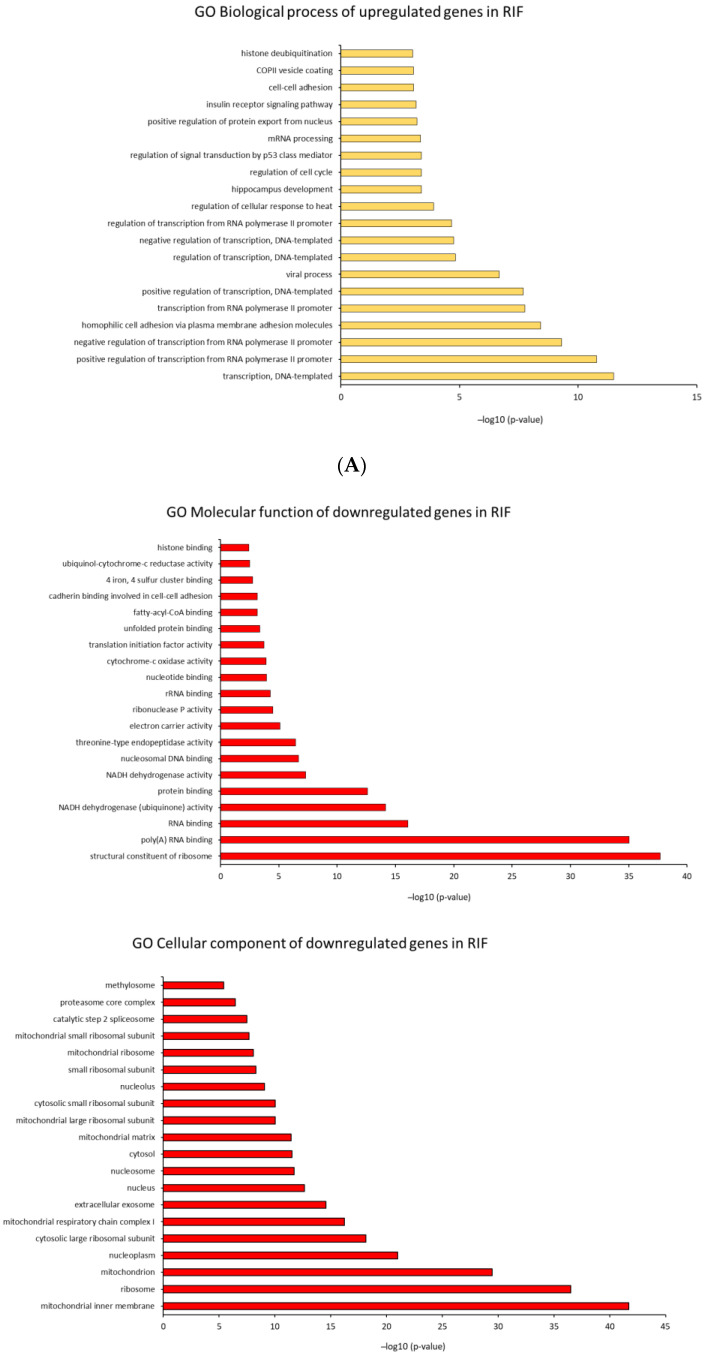

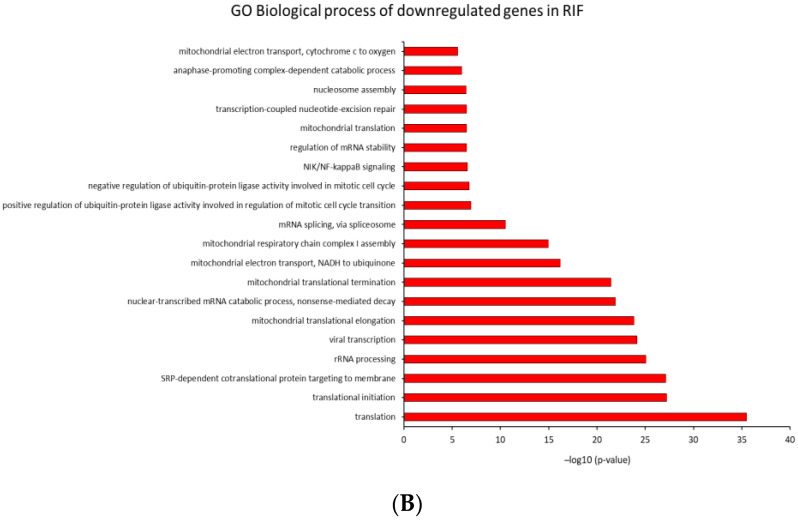
(**A**,**B**) Bioinformatics analysis of DEGs in RIF patients compared to control group of participants. Top 20 up and downregulated Gene Ontology (GO) analyses including GO MF, GO CC and GO BP in RIF patients compared to control group participants with significant *p*-value < 0.05.

**Figure 6:**
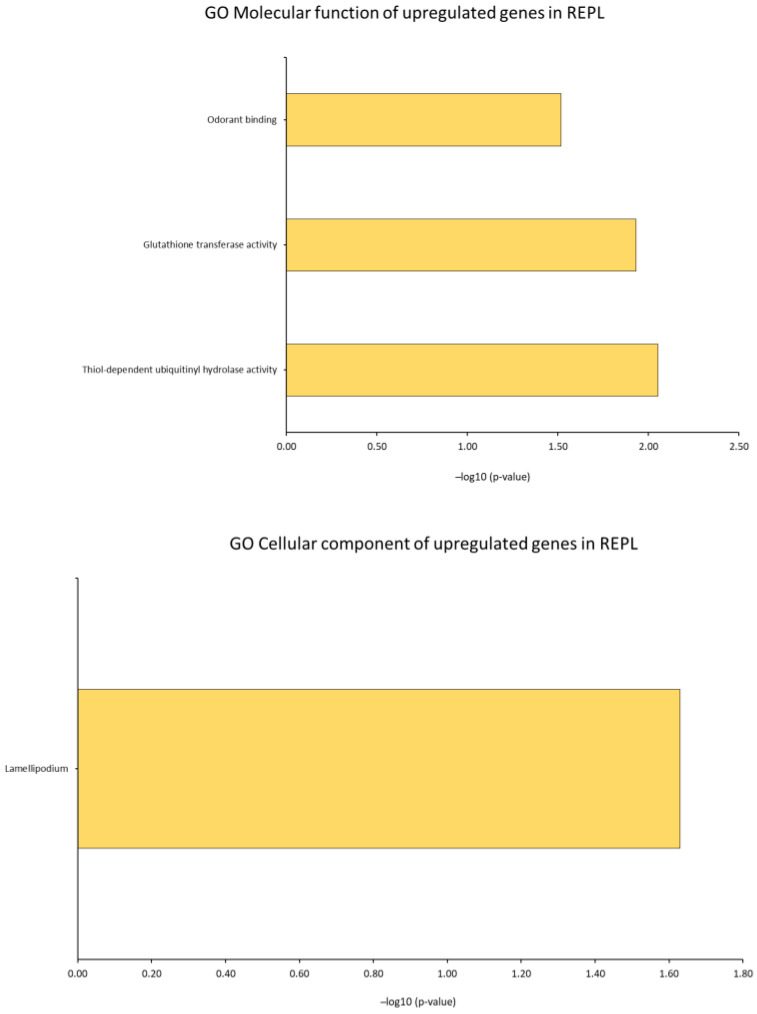

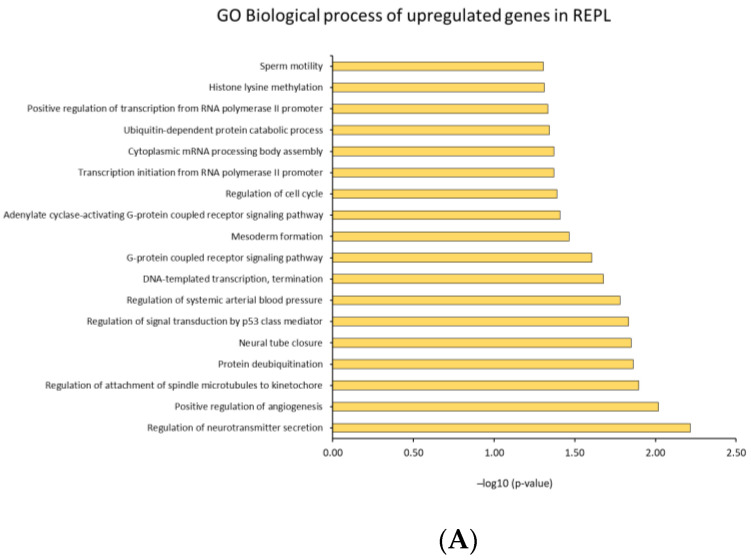

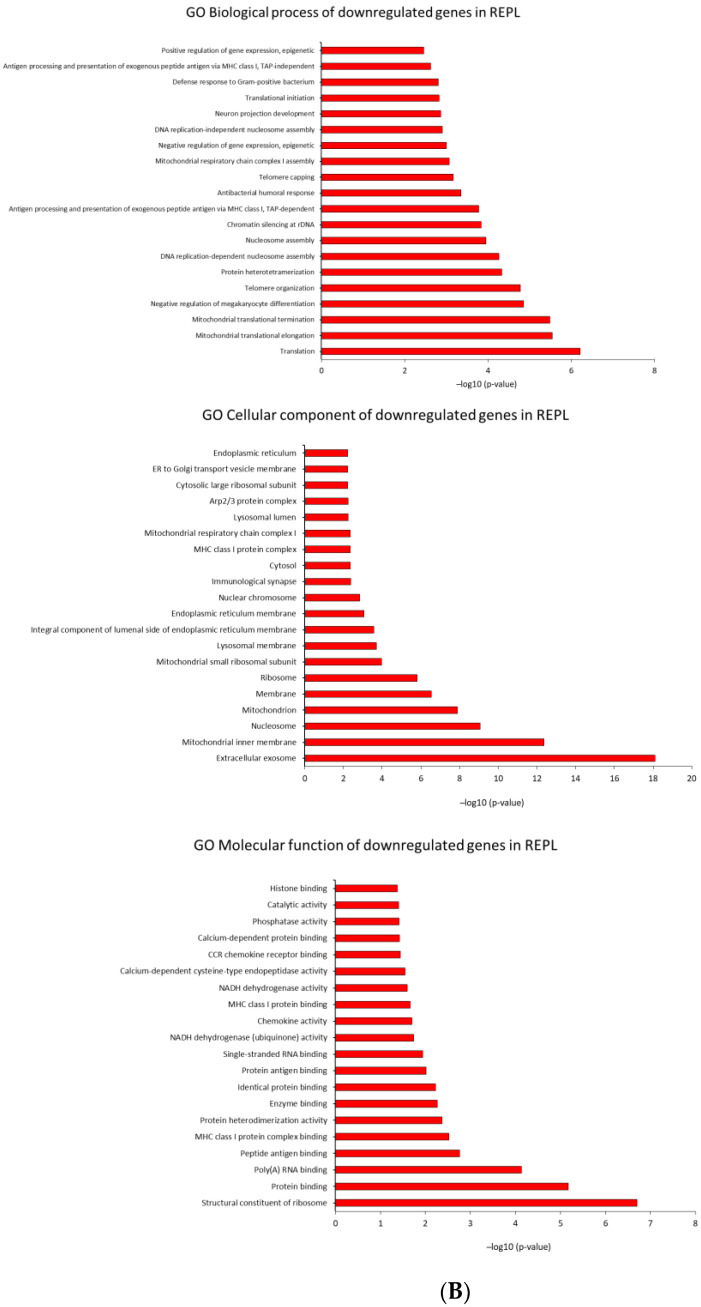
(**A**,**B**) Bioinformatics analysis of DEGs in REPL patients compared to control group participants. Top 20 up and downregulated GO analyses including GO MF, GO CC and GO BP in REPL patients compared to control group participants with significant *p*-value < 0.05.

Meanwhile, for significantly enriched DEGs (*p* < 0.05) of the REPL participants, the GO MF revealed a total number of 26 related functions (3 were upregulated, 23 downregulated). Furthermore, for GO CC, 37 cellular regions were significantly affected (1 upregulated, 36 downregulated), and an obvious change in DEGs for GO BP were observed in REPL participants with 101 processes being affected (19 upregulated, 82 downregulated). These findings proposed that under-expression of multiple molecular activities was closely responsible for REPL. The top 20 of the up and downregulated GO analyses are illustrated in Figure 5 and Figure 6.

## 3. Discussion

Endometrial receptivity is a complex course of events in which the endometrium becomes favourable for embryo implantation under the action of ovarian steroid hormones, during the mid-luteal phase of the ovarian cycle in healthy fertile women [22,23]. Nearly a decade of investigations on the transcriptomic analysis on endometrial receptivity has revealed a comparable difference in the endometrial gene expression profile during the early to mid-luteal phases of the window of implantation (WOI) in fertile women [24]. These observations suggest that molecular changes could be utilized as potential molecular markers of the implantation window, replacing histological evaluation of the endometrium where the accuracy, reproducibility, and its functional significance have been doubted [25,26].

Poor endometrial receptivity, especially during the WOI, is accountable for two-thirds of the reproductive inability such as implantation failure and pregnancy loss, however the process is not fully understood [27]. Microarray analysis of human endometrial biopsies during the mid-luteal phase have provided a broader view on the involvement of DEGs in certain disease states, yet the molecular mechanism of the interactions remains poorly understood [28]. Besides the identification of DEGs, recognizing the enriched pathways is significant for revealing molecular processes altered in RIF and REPL [16].

An understanding of the aberrant mechanisms underlying these conditions could advance the treatment of infertility, ultimately preventing early pregnancy loss and improve pregnancy outcome. In the present study, therefore we aimed to provide a detailed insight into the nature of the biological malfunction and related pathways of DEGs in RIF and REPL as compared to the control group. Further knowledge and investigations on the molecular mechanisms coordinating conditions such as RIF and REPL are needed to elaborate more explicit guidelines and recommendations for clinicians using ARTs while confronting these conditions.

To begin deciphering the molecular changes in RIF and REPL, within this paper, microarray analysis of the Pipelle endometrial biopsy exhibited distinctive difference in the transcriptomic profile intergroup and intragroup in both conditions. A few of the previously conducted studies suggest that abnormalities in the uterine biology that leads to RIF and REPL are due to increased amount of downregulated DEGs [29,30]. However, our findings show an equal possibility (50% up and 50% downregulation) of the DEGs within the endometrium of the patients with the history of RIF and REPL. The contradiction in the findings is possibly due to the heterogeneity in the endometrial receptivity among individuals, the small sample size used in the present study and also may be a feature of the analysis methodology used [31].

Bioinformatic analysis of the DEGs revealed 42 significant pathways (*p* < 0.05) that were correlated to RIF while 22 pathways were revealed in REPL. Interestingly, although distinctive differences in the transcriptomic profile and upregulated biological pathways were observed, clear resemblance was noticed in the KEGG categories of the enriched pathways including downregulation in the pathways related to genetic information processing, human disease, metabolism and organismal systems in both conditions. Among all the enriched pathways in RIF and REPL in our study, ribosome and oxidative phosphorylation pathways seem to be significantly dysregulated in both conditions.

The ribosome, a ribonucleoprotein particle, Is an organelle involved in the synthesis of protein in the cells. Translation of mRNA to protein is vital for cell metabolic processes [32] where cell growth and proliferation were sustained as a result of conversion of essential nutrients into energy and macromolecules [33]. The downregulated ribosomal protein genes clustered in the ribosome pathway in the present study were both cytoplasmic and mitochondrial in origin. The mitochondrial originated protein known as mitochondria ribosome protein gene (MRP) is a protein that is important in the regulation of cellular respiration process besides an alternative role as an apoptosis-inducing factor [34]. In addition, these findings were strengthened by the GO analysis which is also consistent with the downregulation of mitochondrial inner membrane, mitochondria, nucleosome and ribosomes as one of the significantly affected cellular component in both conditions.

Furthermore, in both condition, downregulation in translation and mitochondrial translational elongation were revealed as significantly affected GO biological processes. To the best of our knowledge, there are limited numbers of studies reporting the association between ribosome pathway and disease in RIF and REPL with little explanation. Variation in the number of samples, assay (transcriptomic or proteomic) and sample types (endometrial biopsy, chorionic villi or peripheral blood) between studies complicates further the description of this condition [35,36,37]. In the present study, RNA was extracted directly from the endometrial biopsy of patients with RIF and REPL yet our findings were similar to the one previously reported by Xin et al. that utilized placental chorionic villi from patients experiencing miscarriage to extract the protein. The limitation in the previous study is probably due to the incomplete removal of the decidua from the placental villi [37].

The majority of previous studies on RIF and DEGs utilized endometrial biopsy as the sample for analysis (Supplementary Table S5) while, in the study of REPL, variations in the sample selection were observed (Supplementary Table S6). The development of the placenta is crucial for the maintenance of pregnancy [38]. The basic nuclear and cellular functions of a developing placenta are reported to be shut down through a cascade of events leading to pregnancy loss thus pregnancy loss is regarded as one of the placental-related diseases of pregnancy [35].

Depending on the objective of the study, logically, to investigate the possible changes or status of the endometrium during the mid-luteal phase, in patients with repeated occurrence of pregnancy loss and miscarriage, an endometrial biopsy sample is suggested to be the optimum sample to be collected. Without the interference of the embryo, the pure condition of the endometrium can be revealed. Findings from the present study give us an early overview on the engagement of dysregulation in the mRNA translation activity for protein production in the nucleosome and mitochondrial function in the endometrium of the patients with RIF and REPL history. Therefore, we postulate that, ribosome function was dysregulated in the endometrium of the RIF and REPL patients leading to disruption of mRNA translation activity affecting endometrial cell growth and proliferation hence leading to implantation failure and pregnancy loss.

Considering the role of oxidative phosphorylation pathways, this process is a set of metabolic reaction that convert chemical energy from oxygen molecules or nutrients into adenosine triphosphate (ATP) in both cytosol and mitochondria [39]. Most of the ATP produced by aerobic cellular respiration is made by oxidative phosphorylation. In the present study, downregulation of the oxidative phosphorylation pathway was observed in the patient’s endometrium that had experienced RIF and REPL compared to control groups. There seems to be a close connection between the downregulation of ribosome pathway which involves the MRP genes and the downregulation of oxidative phosphorylation pathway in the present study.

The dysregulated MRP genes were mostly involved in mitochondrial respiratory chain for production of energy needed for cellular function through oxidative phosphorylation. This process take place in the inner mitochondrial membrane [40]. On the other hand, downregulation of several gene sets, encodes with NADH dehydrogenase subunit and Cyclooxygenase (COX) in the present study, have been previously associated with mitochondrial dysfunction [38]. Our findings also revealed dysregulation in the neurodegenerative disease such as in Huntington’s disease, Alzheimer’s disease, and Parkinson’s disease pathways. This pathogenesis has also been associated with cellular respiration and energy metabolism in a previous study, with dysregulation in similar groups of NADH dehydrogenase subunit and Cyclooxygenase (*COX*) encoded genes [41].

Similarly, Lyu et al. previously reported downregulation in gene expression associated with oxidative phosphorylation in the placental villi and showed the involvement of *UQCRB*, a subunit of mitochondrial complex III, A and the *TP5G1* and *ATPG3* genes in the impairment of mitochondrial respiratory chain function in the placental villi was a contributor to miscarriage [38]. However, the genes reported by Lyu et al. were not identified in this present study possibly owing to the differences in the samples being analyzed, and/or it may also reflect a different assay system utilized with the genes being specific to placenta villi.

Recently, proteomic analysis of the decidua in REPL patients reported a significant upregulation on the level of COX-2 and NDUFB3 protein when analyzed by Western blotting. Overexpression of *NDUFB3* has been associated with inhibition of cell vitality and oxidative stress in the decidual cell thus reduction in the mitochondrial membrane potential expression levels indicating the role of *NDUFB3* in promoting the pathogenesis of REPL [42]. In the present study, we found the downregulation of *NDUFB9* and other NADH dehydrogenase encoded genes and *COX6A1*, *COX7A2*, and *COX8A*. These genes were different from the one reported by Yin et al., 2021. Previously, dysregulation in these genes has been associated with mitochondrial disease and dysfunction affecting the energy metabolism that is needed for cell vitality [43].

Finally, in addition to association between MRP and the ribosome and oxidative phosphorylation pathways, during the window of implantation, endometrial glands apoptosis has been reported to be increased to allow successful implantation of the embryo. However, in the present study, downregulation of the *FAU* gene was observed suggesting the inhibition of apoptotic regulation in the endometrium of the RIF and REPL patients during the midluteal phase of the menstrual cycle. Similar findings have been observed in infertile women with endometriosis, tubal factor, and polycystic ovary syndrome [44]. Still, the precise mechanisms that mediates the apoptotic process in normal endometrium and in infertile women is not fully understood. The results obtained warrant further investigations and using larger cohort of individuals and patients to better understand variation between individuals in the population. Further exploration into the mechanism and reasoning of this pathways in both conditions would be beneficial to the overall understanding of the conditions and hence also improve preventative and therapeutic approaches. 

## 4. Materials and Methods

### 4.1. Ethics Approval

Ethical approval was obtained according to the Research Ethics Committee regulations, policies and procedures from University of Sheffield and Sheffield Teaching Hospital NHS Foundation Trust (STH reference number, 18,063).

### 4.2. Patient Selection

This study consisted of three groups of patients categorised as (i) Control group (*n* = 3)—comprises of women with good endometrial receptivity evidenced by the ability to support an ongoing clinical pregnancy after IVF; defined as a confirmed fetal heartbeat detected by ultrasound scanning at 12 weeks of gestation. Women planning IVF treatment were recruited in the menstrual cycle prior to their IVF treatment. Those that achieved clinical ongoing pregnancy were included in the study. (ii) RIF (*n* = 3)—women with failure to achieve a clinical pregnancy following the transfer of four or more good quality embryos in at least three fresh or frozen embryo transfer cycles and (iii) REPL (*n* = 3)—defined as women with three consecutive pregnancy losses prior to 12 weeks from the date of the last menstrual period. A good quality embryo was defined as having the correct number of cells corresponding to the day of its development and day 5 embryos (blastocysts) were graded according to expansion and quality of the inner cell mass and trophoectoderm. Other criteria included blastomeres of equal size and regular in distribution, even distribution of cytoplasm without granularity and less than 10% fragmentation) [45].

### 4.3. Patient Recruitment

Patient recruitment began with the distribution of patient information leaflets during the appointment booking session to the couples who attended the RIF clinic, REPL clinic and Assisted Conception Unit in Jessop Wing, University of Sheffield to ensure they have enough time to read and understand the protocol. During their initial medical consultation, couples who showed interest in participating in the study were approached by a member of the research team (research nurse or consultant/registrar), to further explain the study and answer any questions. Couples who agreed and fulfilled the inclusion/exclusion criteria (Table 1) were recruited into the study and a valid consent was obtained. The couple had the full right to withdraw from the study at any point in time until the endometrial sampling. 

### 4.4. Endometrial Tissue Collection/Biopsy

Pipelle endometrial tissue biopsies and fluid collection were done under ultrasound guidance that was scheduled in the mid-luteal phase of their cycle (7–10 days prior to menstruation, 7 days after ovulation) in the control group, RIF and REPL patients which was judged by a positive LH surge detected on home urine ovulation test. The correct timing was further confirmed by monitoring the number of days to the next period. The sample would be accepted as midluteal if it fulfilled both criteria: 7 days after predicted ovulation and 7–10 days before next menstruation. Barrier contraception were used for the control group on the month of the biopsies collection. For the RIF patient, the procedure was done at the same time of the endometrial scratch procedure routinely offered which constitutes an extremely minor addition to the procedure and carried no individual risk. Biopsies were collected using pipelle. Samples were kept in RNAlater (Thermo Fisher, Loughborough, UK) to preserve the RNA from degenerating and kept in −80 °C until further analysis. 

### 4.5. Total RNA Isolation

RNA was extracted using a combination of Trizol and an affinity column (Zymo Research Corporation, Irvine, CA, USA) with some modifications to the manufacturer’s instructions to maximize yield from small sample. Endometrial biopsies were lysed in TRI Reagent^®^ (Sigma-Aldrich^®^, Dorset, UK). Sample were centrifuged for phase separation and the supernatant were collected. Equal amount of 100% ethanol was added into the supernatant and mixed thoroughly for RNA precipitation. Supernatant was transferred into a Zymo-SpinTM IIC Column in a Collection Tube (Zymo Research Corporation, Irvine, CA, USA) and centrifuged at 12,000× g for 30 s. Samples were DNase I (Qiagen, Düsseldorf, Germany) treated to avoid DNA contamination. Finally, the RNA was eluted with DNase/RNase-Free Water (Sigma-Aldrich^®^, Dorset, UK). The RNA was stored at −80 °C until further used. RNA integrity, purity and concentrations were analysed using Agilent RNA 6000 Pico Kit (Agilent Technologies, Manchester, UK) and measured using an Agilent 2100 G2938B Model B Bioanalyzer (Agilent Technologies, Manchester, UK). 

### 4.6. Microarray Analysis

RNA was converted to cDNA and biotin labelled using the WT plus kit from Affymetrix before being applied to the Affymetrix GeneChip^®^ Human Clariom S Array (Affymetrix, Sunnyvale, CA, USA) with one array being used for each RNA sample. Following hybridization, the arrays were washed and stained on the GeneChip^®^ Fluidics Station 450 (Affymetrix, Sunnyvale, CA, USA) using the appropriate fluidics script, before being inserted into the Affymetrix autoloader carousel and scanned using the GeneChip^®^ Scanner 3000 (Affymetrix, Sunnyvale, CA, USA). All scanned array images were visually inspected for chip surface artefacts that could adversely impact the data.

### 4.7. Bioinformatic Analysis

Data normalisation, differential gene expression analysis and robust statistical calculations were performed using the Transcription Analysis Console (TAC) software version 4.0.1 (Affymetrix, Sunnyvale, CA, USA). Genes were defined as significantly differentially expressed when they meet our pre-set designated criteria of *p* < 0.05 and a greater than 1.2-fold change. Multidimensional scaling plots were generated in Python 3.8.3. Bioinformatic functional analysis of dysregulated genes was explored by using the Database for Annotation, Visualization and Integrated Discovery (DAVID) [46], a gene set-based algorithm that detects functionally related genes in lists of genes ordered according to differential expression. False discovery rate (FDR) of <0.05 was considered statistically significant. All genes identified as statistically differentially expressed were subjected to functional annotation comparisons, gene ontology (GO) and KEGG pathways. In DAVID, functional annotation clustering was employed to interpret the biological meanings of the genes of interest. Enrichment score is used to rank their biological significance. Clusters with enrichment score >1.3 were considered, and the GO-term biological process (BP), molecular function (MF), and cellular component (CC) were examined.

### 4.8. Results Validation

To validate the results generated from the Microarray analysis, data generated were cross examined with published literature to check if the genes that were reported to be significantly altered in literature were also identified in our results, and if they changed in the same pattern. Literature, which were published within the last 20 years, were identified in google scholar, using the following keywords: gene expression profile, endometrium, differentially expressed gene in RIF and REPL, RIF or gene expression profile, REPL or gene expression profile (Supplementary Tables S5 and S6). 

## 5. Conclusions

In conclusion, we have identified, apart from previously reported inflammatory, immune system and infection related pathways in RIF and REPL, alteration in the RNA-protein translation process and energy metabolism pathways. The ribosome and oxidative phosphorylation pathways are significantly downregulated in RIF and REPL. These findings give us an insight to the pathways involved in two different states of reproductive disorder and may thus aid in future diagnostic and therapeutic intervention in preventing recurrent failure in reproductive function. 

## Figures and Tables

**Table 1:** Inclusion and exclusion criteria of the patient’s selection and recruitment in control group, RIF and REPL.

Patient Selection	Control Group (*n* = 3)	RIF (*n* = 3)	REPL (*n* = 3)
Inclusion criteria	Age under 40 years	Age 40 years or less	Age 40 years or less
	Undergoing an IVF cycle that result in an ongoing clinical pregnancy as defined above	Fulfilling the definition of RIF	Fulfilling the definition of REPL
	Consent to participate in this study	Normal results obtained on routine RIF investigations (thrombophilia screening, Karyotyping, hormonal profile, and detailed pelvic scan)	Normal results obtained on routine REPL investigations (thrombophilia screening, Karyotyping, hormonal profile, and detailed pelvic scan)
		Consent to participate in this study	Consent to participation in this study
Exclusion criteria	Women aged 40 years or older	Women aged 40 years or older	Women aged 40 years or older
	Known uterine or endometrial pathology.	Known uterine or endometrial pathology	Known uterine or endometrial pathology
	History of RIF	Known cause of RIF	Known cause of REPL
	Low ovarian reserve defined by a baseline serum FSH level above 10 IU/ML		
	Known uncorrected endocrinological pathologies		

RIF—Recurrent implantation failure, REPL—Recurrent early pregnancy loss.

**Table 2:** Gene symbol, gene name and fold change of the top 20 DEG in RIF.

Gene Symbol	Gene Name	Fold Change	
*MLXIP*	MLX interacting protein	1.53	Upregulated genes
*LRP6*	LDL receptor related protein 6	1.63	
*ZKSCAN7*	zinc finger with KRAB and SCAN domains 7	2.13	
*DAZAP2*	DAZ Associated Protein 2	2.73	
*NRSN2*	neurensin 2	2.77	
*DCAKD*	dephospho-CoA kinase domain containing	2.96	
*ESRP1*	epithelial splicing regulatory protein 1	3.43	
*MAML1*	mastermind-like transcriptional coactivator 1	3.51	
*PROSER1*	proline and serine rich 1	3.57	
*SF3B4*	splicing factor 3b, subunit 4, 49 kDa	3.6	
*SEC24D*	SEC24 Homolog D, COPII Coat Complex Component	3.7	
*MED1*	mediator complex subunit 1	3.87	
*TFG*	TRK-fused gene	5.36	
*NUP214*	nucleoporin 214 kDa	6.15	
*ATXN2*	ataxin 2	8.22	
*TULP3*	tubby like protein 3	10.26	
*CRTC2*	CREB regulated transcription coactivator 2	13.24	
*HLA-DRB1*	major histocompatibility complex, class II, DR beta 1	−5.04	Downregulated genes
*RPS6KA1*	Ribosomal Protein S6 Kinase A1	−2.9	
*GPN3*	GPN-Loop GTPase 3	−2.75	

**Table 3:** Gene symbol, gene name and fold change of the top 20 DEG in REPL.

Gene Symbol	Gene Name	Fold Change	
*SKP2*	Transcript Identified by AceView, Entrez Gene ID(s) 6502	1.9	Upregulated genes
*PLA2G5*	phospholipase A2, group V	1.93	
*MYADML2*	myeloid-associated differentiation marker-like 2	2.08	
*C2orf48*	chromosome 2 open reading frame 48	2.09	
*TMPRSS2*	transmembrane protease, serine 2	2.15	
*MYO18B*	myosin XVIIIB	2.3	
*NKD1*	NKD Inhibitor of WNT Signaling Pathway 1	2.3	
*MAP3K13*	mitogen-activated protein kinase kinase kinase 13	2.38	
*CFAP161*	cilia and flagella associated protein 161	3.33	
*CD96*	CD96 molecule	−6.41	Downregulated genes
*IVNS1ABP*	influenza virus NS1A binding protein	−4.29	
*DDT*	D-dopachrome tautomerase	−3.2	
*RNF114*	ring finger protein 114	−2.91	
*DDTL; DDT*	D-dopachrome tautomerase-like; D-dopachrome tautomerase	−2.65	
*MGAT1*	mannosyl (alpha-1,3-)-glycoprotein beta-1,2-N-acetylglucosaminyltransferase	−2.3	
*HSPB2; C11orf52; HSPB2-C11orf52*	heat shock 27kDa protein 2; chromosome 11 open reading frame 52; HSPB2-C11orf52 readthrough (NMD candidate)	−2.16	
*FAM49B*	family with sequence similarity 49, member B	−1.98	
*ABCC8*	ATP binding cassette subfamily C member 8	−1.98	
*OTUB1*	OTU deubiquitinase, ubiquitin aldehyde binding 1	−1.93	
*RPS6KA1*	ribosomal protein S6 kinase, 90 kDa, polypeptide 1	−1.93	

## Data Availability

Not applicable.

## Supplementary Materials

The supporting information can be downloaded at: https://www.mdpi.com/article/10.3390/ijms232416051/s1.
