# Supplementary material for: Dysregulation in Multiple Transcriptomic Endometrial Pathways Is Associated with Recurrent Implantation Failure and Recurrent Early Pregnancy Loss
Source: Int J Mol Sci. 2022 Dec 16;23(24):16051. doi: 10.3390/ijms232416051 (PMC9782216; doi:10.3390/ijms232416051)
Supplement: Supplementary file 1 [file ijms-23-16051-s001.zip › ijms-1791478-supplementary.pdf]

Supplementary Table S1 Percentage of upregulated genes in top 20 significant KEGG pathways in RIF.

KEGG pathway	Total genes	% of affected genes	Upregulated genes	–log p-value	p-value
**Adherens junction (hsa04520)**	71	18.30%	*TCF7L2, CREBBP, SMAD4, SMAD3, CSNK2A2, PTPRM, WASL, IQGAP1, ACTB, CSNK2B, EP300, WASF2, VCL*	3.37	0.00043
**Transcriptional misregulation in cancer (hsa05202)**	167	12.00%	*SS18, SMAD1, KMT2A, MAX, PRCC, JMJD1C, KLF3, AFF1, SUPT3H, RELA, PTK2, ETV6, RUNX1, ELK4, SPINT1, SP1, SIN3A, BMP2K, RARA, RUNX1T1*	2.71	0.00193
**RNA transport (hsa03013)**	172	11.60%	*NUP214, EIF5B, POM121, NUP153, UPF3A, RGPD2, RGPD1, SRRM1, EIF5, POM121C, XPO5, TPR, SMN2, EIF4EBP2, RNPS1, NUP98, SMN1, EIF4G3, EIF4G2, EIF4G1*	2.57	0.00271
**HTLV-I infection (hsa05166)**	254	10.20%	*RB1, VAC14, ATF2, GSK3B, CRTC2, CRTC3, TRRAP, ADCY2, IL2RG, ELK1, RELA, ELK4, ZFP36, CDC27, EP300, PRKACA, EGR1, CREBBP, SMAD4, RANBP3, SMAD3, TBP, NFATC3, NFATC2, APC, LTBR*	2.51	0.00312
**Glucagon signaling pathway (hsa04922)**	99	13.10%	*ATF2, CREBBP, CRTC2, PRKAB2, ADCY2, ACACA, GYS1, G6PC3, GNAQ, CREB3L2, EP300, PRKACA, CREB5*	2.11	0.007719
**Wnt signaling pathway (hsa04310)**	138	11.60%	*GSK3B, TCF7L2, CREBBP, SMAD4, CHD8, CSNK2A2, NFATC3, NFATC2, NKD1, LRP6, APC, DAAM1, WIF1, CSNK2B, EP300, PRKACA*	2.07	0.00849
**Vasopressin-regulated water reabsorption (hsa04962)**	44	18.20%	*RAB5B, RAB5C, ARHGDIA, CREB3L2, AQP4, PRKACA, VAMP2, CREB5*	2.03	0.00941
**Thyroid hormone signaling pathway (hsa04919)**	115	12.20%	*NCOA1, NCOA2, GSK3B, MED1, CREBBP, HIF1A, ACTB, MED13L, MED14, MED13, TBC1D4, SIN3A, EP300, PRKACA*	1.996	0.01009
**Ubiquitin mediated proteolysis (hsa04120)**	137	10.90%	*PIAS3, CUL5, FBXW8, UBA7, UBE2D3, HUWE1, SYVN1, UBE4A, PIAS1, RHOBTB2, UBE2Q1, UBE2R2, CDC27, UBE2U, BIRC2*	1.75	0.01790
**Thyroid cancer (hsa05216)**	29	20.70%	*RET, TCF7L2, TFG, TPM3, TPR, CCDC6*	1.71	0.01944
**Vibrio cholerae infection (hsa05110)**	52	15.40%	*KDELR1, ATP6V0A2, KDELR2, KDELR3, PRKACA, ATP6V0C, ACTB, ATP6V0A1*	1.65	0.02259
**Pathways in cancer (hsa05200)**	393	8.10%	*RET, RB1, GSK3B, MAX, PTGER3, ADCY2, HIF1A, RELA, GLI3, EDNRA, GNG2, TFG, TPR, FGF20, EP300, PRKACA, RUNX1T1,*	1.59	0.02553

			TCF7L2, CREBBP, SMAD4, SMAD3, TPM3, VEGFC, PTK2, RUNX1, GNG13, APC, GNAQ, RARA, CCDC6, SOS1, BIRC2		
**Hepatitis B (hsa05161)**	145	10.30%	*RB1, ATF2, CREBBP, SMAD4, IFNA14, ATF6B, NFATC3, NFATC2, TICAM1, ELK1, RELA, MAVS, CREB3L2, EP300, CREB5*	1.56	0.02778
**Insulin resistance (hsa04931)**	108	11.10%	*MLXIP, GSK3B, GYS1, CRTC2, PRKAB2, G6PC3, TBC1D4, PPP1R3E, CREB3L2, PPP1R3A, RELA, CREB5*	1.46	0.03433
**Cell cycle (hsa04110)**	124	10.40%	*RB1, YWHAE, GSK3B, CREBBP, SMAD4, SMAD3, SMC1A, RBL2, WEE1, ORC3, CDC27, EP300, E2F5*	1.41	0.03922
**Dopaminergic synapse (hsa04728)**	128	10.20%	*ATF2, GSK3B, GSK3A, ATF6B, CACNA1A, GNG13, GNAL, GNG2, KIF5B, GNAQ, CREB3L2, PRKACA, CREB5*	1.32	0.04814
**NK-KAPPA B Signaling pathway (hsa04064)**	87	11.50%	*CSNK2A2, CSNK2B, TAB3, TRIM25, TAB2, LTBR, ERC1, TICAM1, RELA, BIRC2*	1.31	0.04848

Supplementary Table S2 Percentage of downregulated genes in top 20 significant KEGG pathways in RIF.

KEGG pathway	Total genes	% of affected genes	Downregulated genes	–log p-value	p-value
**Ribosome (hsa03010)**	136	50%	*MRPS17, RPL3, RPL32, RPL31, MRPS14, RPL34, RPLP0, MRPS12, MRPS10, MRPL36, MRPL34, RPL8, RPL9, RPL6, RPL7, RPS4X, RPS15, RPS17, MRPL2, RPS19, RPL36AL, RPL35, RPL38, RPL37, MRPL9, RPS13, RPS12, RPS9, RPL21, RPL36A-HNRNPH2, RPL23, RPS6, MRPS18A, MRPS21, RPS3A, RPSA, MRPS6, RPL37A, RPL24, RPL27, UBA52, RPL11, MRPL17, RPS27L, MRPL14, MRPL13, MRPL11, MRPL20, RPS15A, RPL14, RPS2, RPS27A, RPL19, RPL41, MRPL27, RPL23A, MRPL23, MRPL24, MRPL22, MRPL30, RPS25, RPL27A, FAU, RPS21, RSL24D1, RPL26L1, RPS24, RPS23*	36.93	0.0000
**Parkinson's disease (hsa05012)**	142	35.90%	*NDUFA13, COX7B, NDUFA11, NDUFA12, COX4I1, NDUFA10, ATP5G3, PARK7, ATP5G2, COX6A1, ATP5G1, COX7C, CASP9, UQCRFS1, COX8A, NDUFC1, SDHD, SDHB, COX7A2L, NDUFS8, NDUFS7, NDUFS6, NDUFS4, NDUFS3, PPIF, NDUFS2, SLC25A5, NDUFB9, NDUFB8, NDUFB10, NDUFB5, ATP5A1, NDUFB2, NDUFB1, UQCR10, COX5B, COX5A, UQCRH, UBB, NDUFV1, NDUFA9, NDUFA8, NDUFA6, NDUFA5, NDUFA4, NDUFA2, NDUFA1, COX6C, UQCRHL, PINK1, UQCRQ*	19.50	0.0000
**Huntington's disease (hsa05016)**	192	30.70%	*NDUFA13, COX7B, NDUFA11, NDUFA12, COX4I1, NDUFA10, CLTB, ATP5G3, ATP5G2, COX6A1, ATP5G1, COX7C, CASP9, UQCRFS1, IFT57, TGM2, COX8A, NDUFC1, SDHD, SDHB, COX7A2L, NDUFS8, NDUFS7, NDUFS6, NDUFS4, NDUFS3, PPIF, TBPL1, TFAM, NDUFS2, SLC25A5, NDUFB9, NDUFB8, HDAC2, NDUFB10, NDUFB5, ATP5A1, NDUFB2, NDUFB1, UQCR10, COX5B, COX5A, UQCRH, AP2S1, POLR2F, POLR2I, NDUFV1, NDUFA9, NDUFA8, NDUFA6, NDUFA5, NDUFA4, NDUFA2, NDUFA1, COX6C, UQCRHL, SOD1, UQCRQ, TAF4B*	18.87	0.0000
**Oxidative phosphorylation (hsa00190)**	133	36.10%	*NDUFB9, COX7B, NDUFA13, NDUFB8, NDUFB10, NDUFA11, NDUFA12, NDUFB5, COX4I1, NDUFA10, ATP5A1, NDUFB2, NDUFB1, ATP5I, ATP5G3, UQCR10, ATP5G2, COX5B, COX6A1, ATP5G1, COX7C, COX5A, UQCRH, UQCRFS1, NDUFV1, ATP6V1D,*	18.42	0.0000

			COX8A, NDUFA9, NDUFA8, NDUFA6, NDUFA5, NDUFA4, NDUFA2, NDUFA1, NDUFC1, SDHD, COX6C, SDHB, UQCRHL, COX7A2L, NDUFS8, NDUFS7, PPA2, UQCRQ, NDUFS6, NDUFS4, NDUFS3, NDUFS2		
**Alzheimer's disease (hsa05010)**	168	31.50%	*NDUFA13, COX7B, NDUFA11, NDUFA12, COX4I1, NDUFA10, ATP5G3, ATP5G2, COX6A1, ATP5G1, COX7C, CASP9, UQCRFS1, COX8A, NDUFC1, SDHD, SDHB, COX7A2L, NDUFS8, NDUFS7, NDUFS6, NDUFS4, NDUFS3, NDUFS2, NDUFB9, NDUFB8, NDUFB10, NDUFB5, ATP5A1, NDUFB2, NDUFB1, UQCR10, COX5B, COX5A, HSD17B10, UQCRH, NAE1, FADD, APOE, BID, NDUFV1, NDUFA9, NDUFA8, NDUFA6, NDUFA5, NDUFA4, NDUFA2, NDUFA1, COX6C, UQCRHL, CDK5, UQCRQ, CALM1*	17.45	0.0000
**Non-alcoholic fatty liver disease (NAFLD) (hsa04932)**	151	29.10%	*NDUFB9, COX7B, NDUFA13, NDUFB8, NDUFB10, NDUFA11, NDUFA12, NDUFB5, COX4I1, NDUFA10, NDUFB2, NDUFB1, UQCR10, COX5B, COX6A1, COX7C, COX5A, UQCRH, UQCRFS1, BID, NDUFV1, COX8A, NDUFA9, NDUFA8, NDUFA6, NDUFA5, NDUFA4, NDUFA2, NDUFA1, NDUFC1, SDHD, COX6C, SDHB, UQCRHL, NFKB1, COX7A2L, NDUFS8, NDUFS7, UQCRQ, NDUFS6, DDIT3, NDUFS4, NDUFS3, NDUFS2*	12.98	0.0000
**Spliceosome (hsa03040)**	133	26.30%	*ISY1, DDX5, RBM8A, SF3B6, SRSF1, SNU13, U2AF1, PQBP1, SNRPD2, ZMAT2, SNRPD3, CTNNBL1, ISY1-RAB43, PPIL1, NCBP2, BUD31, ALYREF, THOC1, PLRG1, CWC15, LSM8, LSM7, PRPF6, LSM6, SYF2, SNRPG, PPIE, PPIH, SNRPA1, SNRPE, SNRPF, SNRPC, HSPA1B, HSPA1A, SRSF9*	8.98	0.0000
**Systemic lupus erythematosus (hsa05322)**	134	23.10%	*HIST1H2BO, HIST1H2BJ, HIST1H2BL, HIST1H2BK, HIST1H3A, H2AFJ, HIST1H2AE, HIST1H3F, HIST1H2AG, SNRPD3, HIST1H3B, HIST1H2AB, HIST1H3E, HIST1H2AI, H3F3B, H2AFZ, H3F3C, HIST1H4L, HIST1H2AH, HIST1H2AJ, HIST2H3A, HIST4H4, HIST1H2BF, HLA-DPB1, HIST1H4H, HIST1H2BH, HIST1H2BG, HIST2H3C, HIST1H2BD, HIST1H4E, HLA-DRB1*	6.59	0.0000
**RNA transport (hsa03013)**	172	20.90%	*EIF4A2, CYFIP1, POP5, EIF4A1, SEH1L, POP7, RBM8A, NUP188, POP1, GEMIN2, RPP30, NMD3, NUP160, RPP14, PAIP1, PRMT5, EIF2B3, NCBP2, ALYREF, ELAC1, RPP40, THOC1, PYM1, TRNT1, THOC7, SNUPN, RPP25L, THOC6, EIF2S3, RPP21, EIF3G, NUP35, GEMIN6, EIF3F, EIF4E2, RAN*	6.50	0.0000
**Alcoholism (hsa05034)**	177	18.60%	*HDAC2, HIST1H2BO, HIST1H2BJ,*	4.85	0.0000

			HIST1H2BL, HDAC11, HIST1H2BK, HDAC8, HIST1H3A, H2AFJ, HIST1H2AE, HIST1H3F, HIST1H2AG, HIST1H3B, HIST1H2AB, HIST1H3E, HIST1H2AI, H3F3B, H2AFZ, H3F3C, HIST1H4L, HIST1H2AH, HIST1H2AJ, HIST2H3A, HIST4H4, HIST1H2BF, HIST1H4H, GRB2, HIST1H2BH, HIST1H2BG, CALM1, HIST2H3C, HIST1H2BD, HIST1H4E		
**Metabolic pathways (hsa01100)**	1219	11.10%	*NDUFA13, PANK2, NDUFA11, TUSC3, NDUFA12, NDUFA10, SAT2, ACSM4, COX6A1, LIPF, SCP2, UROD, FPGT, ACAA1, PHYKPL, HOGA1, ALG1, SDHD, PGD, ALG10, SDHB, DCTD, MTAP, HMBS, SUCLG2, SUCLG1, LAP3, MTMR3, NDUFB10, ATP5I, LIAS, ADH5, UQCRH, NME1-NME2, PPCS, PMVK, PGK1, NDUFV1, ATP6V1D, FDPS, GGT7, PDHA1, PRDX6, CRLS1, COQ7, POLA2, UQCRQ, ADI1, ITPA, COX7B, PIGN, COX4I1, ATP5G3, ATP5G2, ATP5G1, COX7C, PIGY, HK1, SPTLC1, ZNRD1, GUK1, CD38, UQCRFS1, PGLS, COX8A, ACAD8, CERS5, TPI1, PGAM1, NME2, NME3, NDUFC1, DDOST, APRT, NME1, NT5C3A, PIGC, UGDH, NDUFS8, NDUFS7, TST, PCCA, ACOX1, NDUFS6, IVD, DPYD, NDUFS4, ST20-MTHFS, NDUFS3, NDUFS2, PGAM4, MAN1B1, TKT, B4GALT7, DLD, UGT2B7, ALDH9A1, NDUFB9, NDUFB8, NDUFB5, RPE, ATP5A1, NDUFB2, NDUFB1, HSD17B4, MAT2B, UQCR10, COX5B, AGPAT2, COX5A, HSD17B10, HMGCL, UGP2, CBS, POLR2F, BPNT1, MGAT1, POLR2I, CDIPT, NDUFA9, NDUFA8, NDUFA6, NDUFA5, MDH1, NDUFA4, PTGES3, NDUFA2, NDUFA1, COX6C, TRIT1, UQCRHL, PFKL, GNPDA1, IMPDH2, POLR3H*	4.042	0.0001
**Proteasome (hsa03050)**	44	29.50%	*PSMB8, PSMB10, PSMA5, PSMB6, PSMA6, PSMB7, PSMB4, PSMC6, PSMA4, PSMB5, PSMD4, PSMA1, PSMB3*	3.82	0.0002
**Ribosome biogenesis in eukaryotes (hsa03008)**	87	19.50%	*POP5, POP7, POP1, RPP30, RPP40, FCF1, SNU13, NMD3, RPP25L, RRP7A, FBL, EMG1, LOC81691, EIF6, NHP2, RAN, NOP10*	2.78	0.00165
**Protein export (hsa03060)**	23	34.80%	*IMMP1L, SPCS1, SEC61G, SRP54, SEC61B, SRP14, SEC11A, SRP9*	2.74	0.00184
**Viral carcinogenesis (hsa05203)**	205	14.60%	*HDAC2, HIST1H2BO, HIST1H2BJ, TRADD, GTF2B, HIST1H2BL, HDAC11, HIST1H2BK, CHD4, UBE3A, HDAC8, HIST1H4L, MRPS18B, SCRIB, HLA-G, NFKB1, NFKB2, HLA-E, CCNE2, HIST4H4, CDK4, HIST1H2BF, CDK1, HIST1H4H, TBPL1, GRB2, HIST1H2BH, HIST1H2BG, HIST1H2BD, HIST1H4E*	2.61	0.00247
**Biosynthesis of**	212	14.20%	*RPE, HSD17B10, ADH5, HK1, NME1-NME2,*	2.39	0.00407

**antibiotics (hsa01130)**			*UGP2, CBS, PGK1, BPNT1, PGLS, ACAA1, FDPS, PDHA1, TPI1, MDH1, PGAM1, NME2, NME3, SDHD, PGD, SDHB, NME1, PFKL, PCCA, SUCLG2, PGAM4, SUCLG1, TKT, DLD, ALDH9A1*		
**Carbon metabolism (hsa01200)**	113	16.80%	*PDHA1, TPI1, MDH1, PGAM1, RPE, SDHD, PGD, SDHB, ADH5, HK1, PFKL, PCCA, PGK1, SUCLG2, PGAM4, SUCLG1, PGLS, TKT, DLD*	2.34	0.00455
**Cardiac muscle contraction (hsa04260)**	75	18.70%	*COX8A, COX7B, COX4I1, UQCR10, COX6C, COX5B, COX6A1, COX5A, COX7C, UQCRHL, UQCRH, COX7A2L, UQCRQ, UQCRFS1*	2.13	0.00734
**Legionellosis (hsa05134)**	54	20.40%	*EEF1G, CASP9, HBS1L, ITGB2, IL18, NFKB1, HSPA1B, HSPD1, NFKB2, HSPA1A, TLR2*	1.95	0.01123
**DNA replication (hsa03030)**	36	22.20%	*POLA2, RNASEH2A, PCNA, RFC2, RPA1, RPA2, SSBP1, MCM6*	1.61	0.02473

Supplementary Table S3 Percentage of upregulated genes in top 20 significant KEGG pathways in REPL.

KEGG pathway	Total genes	% of affected genes	Upregulated genes	–log p-value	p-value
**Lysine degradation (hsa00310)**	52	11.50%	*SETD7, WHSC1L1, AADAT, COLGALT2, EHMT2, HYKK*	2.06	0.008632
**Metabolism of xenobiotics by cytochrome P450 (hsa00980)**	74	9.50%	*UGT2B15, UGT2B17, AKR1C2, CYP2D6, GSTM5, GSTT2, GSTT2B*	2.03	0.009388
**Drug metabolism - cytochrome P450 (hsa00982)**	68	8.80%	*UGT2B15, UGT2B17, CYP2D6, GSTM5, GSTT2, GSTT2B*	1.60	0.025347

Supplementary Table S4 Percentage of downregulated genes in top 20 significant KEGG pathways in REPL.

KEGG pathway	Total genes	% of affected genes	Downregulated genes	–log p-value	p-value
**Antigen processing and presentation (hsa04612)**	76	22.40%	*CREB1, CTSB, HSPA1A, HSPA1B, KLRC1, KLRC2, KLRC3, KLRC4, KLRD1, LGMN, HLA-A, HLA-C, HLA-E, HLA-G, HLA-DPB1, HLA-DRB1, PSME2*	7.16	0.00000
**Systemic lupus erythematosus (hsa05322)**	134	14.90%	*H2AFJ, C1S, HIST1H2AD, HIST1H2AE, HIST1H2AG, HIST1H2AK, HIST1H2BD, HIST1H2BJ, HIST1H2BK, HIST1H2BL, HIST1H3A, HIST1H3D, HIST1H4I, HIST1H4J, HIST1H4K, HIST1H4L, HIST2H4A, HIST2H4B, HLA-DPB1, HLA-DRB1*	5.57	0.00000
**Alcoholism (hsa05034)**	177	11.90%	*H2AFJ, CREB1, CALM1, HIST1H2AD, HIST1H2AE, HIST1H2AG, HIST1H2AK, HIST1H2BD, HIST1H2BJ, HIST1H2BK, HIST1H2BL, HIST1H3A, HIST1H3D, HIST1H4I, HIST1H4J, HIST1H4K, HIST1H4L, HIST2H4A, HIST2H4B, HDAC11, PPP1CA*	4.33	0.00005
**Ribosome (hsa03010)**	136	13.20%	*FAU, MRPL11, MRPL14, MRPL22, MRPL27, MRPL9, MRPS12, MRPS17, MRPS5, RPL13, RPL19, RPL27A, RPL35, RPL36AL, RPS15, RPS9, RPLP0, UBA52*	4.31	0.00005
**Alzheimer's disease (hsa05010)**	168	10.70%	*NDUFS2, NDUFV1, NDUFA1, NDUFA11, NDUFA9, NDUFB2, NDUFB9, APOE, CALM1, CAPN1, COX6A1, COX7A2, COX8A, PSEN1, SDHB, SDHD, UQCRQ, UQCRFS1*	3.19	0.00065
**Oxidative phosphorylation (hsa00190)**	133	11.30%	*ATP5I, NDUFS2, NDUFV1, NDUFA1, NDUFA11, NDUFA9, NDUFB2, NDUFB9, COX6A1, COX7A2, COX8A, SDHB, SDHD, UQCRQ, UQCRFS1*	2.88	0.00132
**Non-alcoholic fatty liver disease**	151	10.60%	*BAX, FASLG, NDUFS2, NDUFV1, NDUFA1, NDUFA11, NDUFA9, NDUFB2, NDUFB9, COX6A1,*	2.79	0.00162

**(NAFLD) (hsa04932)**			*COX7A2, COX8A, SDHB, SDHD, UQCRQ, UQCRFS1*		
**Graft-versus-host disease (hsa05332)**	33	21.20%	*FASLG, HLA-A, HLA-C, HLA-E, HLA-G, HLA-DPB1, HLA-DRB1*	2.65	0.00221
**Viral carcinogenesis (hsa05203)**	205	9.30%	*BAX, CREB1, HIST1H2BD, HIST1H2BJ, HIST1H2BK, HIST1H2BL, HIST1H4I, HIST1H4J, HIST1H4K, HIST1H4L, HIST2H4A, HIST2H4B, HDAC11, HLA-A, HLA-C, HLA-E, HLA-G, MRPS18B, SCRIB*	2.63	0.00235
**Allograft rejection (hsa05330)**	37	18.90%	*FASLG, HLA-A, HLA-C, HLA-E, HLA-G, HLA-DPB1, HLA-DRB1*	2.39	0.00404
**Endocytosis (hsa04144)**	241	8.30%	*RAB35, ARPC1B, ARPC2, ARPC5, ARRB1, CHMP2A, CHMP4B, FGFR2, HSPA1A, HSPA1B, IL2RB, KIF5A, HLA-A, HLA-C, HLA-E, HLA-G, NEDD4L, PARD6B, SNX12, SNX4*	2.23	0.00594
**Parkinson's disease (hsa05012)**	142	9.90%	*NDUFS2, NDUFV1, NDUFA1, NDUFA11, NDUFA9, NDUFB2, NDUFB9, COX6A1, COX7A2, COX8A, SDHB, SDHD, UQCRQ, UQCRFS1*	2.19	0.00652
**Huntington's disease (hsa05016)**	192	8.90%	*BAX, NDUFS2, NDUFV1, NDUFA1, NDUFA11, NDUFA9, NDUFB2, NDUFB9, POLR2G, CREB1, COX6A1, COX7A2, COX8A, SDHB, SDHD, UQCRQ, UQCRFS1*	2.17	0.00669
**Type I diabetes mellitus (hsa04940)**	42	16.70%	*FASLG, HLA-A, HLA-C, HLA-E, HLA-G, HLA-DPB1, HLA-DRB1*	2.12	0.00765
**Viral myocarditis (hsa05416)**	57	14%	*ITGB2, HLA-A, HLA-C, HLA-E, HLA-G, HLA-DPB1, HLA-DRB1, RAC3*	2.03	0.00937
**Autoimmune thyroid disease (hsa05320)**	52	13.50%	*FASLG, HLA-A, HLA-C, HLA-E, HLA-G, HLA-DPB1, HLA-DRB1*	1.68	0.02096
**Spliceosome (hsa03040)**	133	9%	*ISY1, LSM7, U2AF1, HSPA1A, HSPA1B, PQBP1, PRPF31, PRPF6, SNRPD2, SNRPC, SF3B5, ZMAT2*	1.62	0.02378
**Lysosome (hsa04142)**	121	9.10%	*NAGLU, ASAH1, NPC2, CTSA, CTSB, CTSW, GGA2, LGMN, NEU1, SMPD1, SUMF1*	1.52	0.03049

**Proteasome (hsa03050)**	44	13.60%	*PSMC3, PSMC6, PSMD4, PSME2, PSMB10, PSMB3*	1.44	0.03599

Supplementary Table S5 Previous study reporting the genes and pathways associated with RIF

	Author	Sampled used	Analysis method	Assay	Summary of the findings	
					(Main findings) Dysregulated function / pathways in REPL	Genes / protein associated with dysregulation
**1.**	Tapia et al., 2008 [1]	Endometrial biopsy	cDNA microarrays	Transcriptomic	↓ complement system	↓ ↑ *MMP-7*, *CXCR4*, *PAEP* and *C4BPA*
**2.**	Koler et al., 2009 [2]	Endometrial biopsies	Gene array, western blot	Transcriptomic	↓ cell cycle, Wnt signaling and cellular adhesion pathways	↓ cyclin E2, *SFRP1* and *LEF1*<br><br>↑ Slug and *DKK1*
**3.**	Lédée et al., 2011 [3]	Endometrial biopsy	Affymetrix microarray	Transcriptomic	↑ ↓ deregulated cellular function corresponds to cell morphology, cellular development, cell cycle, and cellular assembly.<br><br>↑ highly deregulated cell-mediated immune response and nervous system development and function.	-
**4.**	Revel et al., 2011 [4]	Endometrial biopsy	TaqMan miRNA arrays cards	Transcriptomic	Ω enriched adherens junctions, cell adhesion molecules,<br>Wnt-signaling, p53 signaling and cell cycle pathways.	↓ mRNA levels of N-cadherin, *H2AFX*, netrin-4 and secreted frizzled-related protein-4, belonging to the cell adhesion molecules, Wnt signaling and cell cycle pathways

	Author	Sampled used	Analysis method	Assay	Summary of the findings	
					(Main findings)<br>Dysregulated function / pathways in REPL	Genes / protein associated with dysregulation
**5.**	Manohar et al., 2014 [5]	Endometrial biopsies	liquid chromatography-mass spectrometric analysis (LC-MS)	Proteomic	-	↑ expression of Ras-related protein Rap-1b, Protein disulfide isomerase A3, Apolipoprotein-A1 (Apo-A1), Cofilin-1 and RAN GTP-binding nuclear protein (Ran)<br><br>↓ Tubulin polymerization promoting protein family member 3, Superoxide dismutase [Cu-Zn], Sorcin, and Proteasome subunit alpha type-5
**6.**	Koot et al., 2016 [6]	Endometrial biopsy	Microarray hybridization	Transcriptomic	↓ cellular proliferation.<br>↓ down-regulation in RIF patients of genes involved in cell cycle regulation and cell division<br><br>↓ expression of many genes involved in general proliferative processes and cytoskeleton and cilia formation<br><br>↑ extracellular organization and cell motility	-
**7.**	Choi et al., 2016 [7]	Endometrial biopsy	Microarray	Transcriptomic	↓ ↑ Leukemia inhibitory factor (LIF) signaling and a P4 response<br><br>≠ Estrogen receptor α (ERα) and Progesterone receptor (PR)	↓ S100 calcium binding protein P (S100P), Chemokine (C-X-C motif) ligand 13 (CXCL13) and SIX homeobox 1 (SIX1) in RIFE

					↓ of expression and phosphorylation of Signal transducer and activator of tran scription 3 (STAT3) and a gene set associated with Janus kinase (JAK)-STAT signaling pathway	

	**Author**	**Sampled used**	**Analysis method**	**Assay**	**Summary of the findings** (Main findings) Dysregulated function / pathways in REPL	**Summary of the findings** Genes / protein associated with dysregulation
**8.**	Shi et al., 2017 [8]	Endometrial biopsy	miRNA microarray	Transcriptomic	↓ ↑ dysregulated miRNAs were miR-30 family, human embryonic stem cell regulation, epithelial-mesenchymal transition, and miRNA tumor suppressors by tool for annotations of microRNA analysis	has-miR-4668-5p, has-miR-429, has-miR-5088
**9.**	Huang et al., 2017 [9]	Endometrial biopsy	RNA sequencing	Transcriptomic	↑ Complementary and coagulation cascades pathway	↑ C3, C4, C4BP, DAF, DF and *SERPING1*.
**10.**	Maekawa et al., 2017 [10]	Samples of endometrial curetting	Affymetrix microarray	Transcriptomic	↑ genes in the thin endometrium related to immunity processes, such as the “response to external stimulus,” “defense response,” “leukocyte mediated immunity,” “immune response,” “immune effector process,” and “regulation of immune system process<br><br>↓ butanoate metabolism in the thin endometrium	↑ Genes related to oxidation-reduction PPAR-γ, *XDH*, *CBR3*, *IDH1*, and *CPT1*, which have essential roles in the cellular responses to oxidative stress<br><br>↓ *CPT1*, *HMGCS2*, and *OXCT1* essential for generating acetyl-CoA and ketone bodies in butanoate metabolism
**11.**	Demiral et al., 2017 [11]	Endometrial biopsies	Microarray	Transcriptomic	The three most highly ranked pathways were Adherens junction, Shigellosis and Cell cycle pathways.	↑ genes in common in the Adherens Junction and Shigellosis pathways: *ACTB* and *WASF1*.
**12.**	Bastu et al., 2018 [12]	Endometrial biopsy	Microarray	Transcriptomic	Pathways enriched include: circadian rhythm, pathways in cancer, proteasome, complement	

	Author	Sampled used	Analysis method	Assay	Summary of the findings (Main findings) Dysregulated function / pathways in REPL	Genes / protein associated with dysregulation
					and coagulation cascades, citrate cycle, adherens junction, immune system and inflammation, cell cycle, and renin–angiotensin system.	-
**13.**	Shi et al., 2018 [13]	Endometrial biopsies	Microarray	Transcriptomic	↓ cytokine-cytokine receptor interaction, p53 signalling and the complement and coagulation cascades pathways.<br><br>↑ PPAR signalling, hematopoietic cell lineage, phosphatidylinositol signalling system, ECM-receptor interaction and notch signalling pathways.	↓ *AQP3*, *DPP4* and *TIMP3*
**14.**	Wang et al., 2019 [14]	Endometrial gene expression profiles of RIF women from National Center for Biotechnology Information (NCBI) Gene Expression Omnibus	Functional enrichment analysis	Transcriptomic	↑ DEGs were significantly enriched in Taste transduction, Olfactory transduction, Neuroactive ligand-receptor interaction, Rap1 signaling pathway, Rheumatoid arthritis, and Tryptophan metabolism.<br><br>↓ DEGs were enriched in Thyroid cancer and RNA transport	Enriched UBE2I, *PLK4*, *XPO1*, *AURKB*, and *NUP107*
**15.**	Gao et al., 2020 [15]	Human endometrial microarray data of RIF and normal control group were obtained from the GEO database provided by the National Center	Bioinformatics analysis	Transcriptomic	Top three related KEGG pathways were cancer pathway, MAPK signaling pathway, and homologous recombination pathway	↓ *PAEP*, *CXCL14*, *HOXB3*, *CD55*, and *VEGFA*

	Author	Sampled used	Analysis method	Assay	Summary of the findings (Main findings) Dysregulated function / pathways in REPL	Genes / protein associated with dysregulation
		for Biotechnology Information (NCBI)				
**16.**	Wang et al., 2021 [16]	Endometrial tissue collection	LC-MS/MS	Proteomic	↓ GOs analysis - regulation of hydrolase activity, blood microparticle, and enzyme inhibitor activity through hypergeometric testing.<br><br>KEGG pathway mapping revealed the immune system (seven proteins), transport and catabolism (five proteins), and translation (five proteins) pathways.<br><br>Enriched KEGG pathway analysis showed the ribosome and primary immunodeficiency pathways as significant with $P < 0.05$	-

↑ upregulated, ↓ downregulated, ↓ ↑ dysregulated/deregulated, Ω enriched, ≠ not significantly altered

Supplementary Table S6 Previous study reporting the genes and pathways associated with REPL.

	Author	Sampled used	Analysis method	Assay	Summary of the findings	
					**(Main findings)<br>Dysregulated function / pathways in REPL**	**Genes / protein associated with dysregulation**
**1.**	Lee at el., 2007 [17]	Endometrial biopsy	qRT-PCR	Genomic	Dysregulation in implantation related gene:<br>↑ transcript levels of cellular retinoic acid binding protein 2 and olfactomedin 1<br><br>↓ Complement component 4 binding protein alpha	↑ *CRABP2*, *OLFM1*<br>↓ *C4BPA*
**2.**	Lédée et al., 2011 [3]	Endometrial biopsy	Affymetrix microarray	Transcriptomic	↓ ↑ deregulated cellular functions relate to cell signalling (degradation of cyclic AMP and calcium metabolism) and cellular maintenance.<br><br>over-representation of deregulations related to the haematological system.<br><br>↓ ↑ deregulations are in organ and tissue development, humoral immune response, and muscular system development and function	-
**3.**	Othman et al., 2012 [18]	Endometrial biopsy	Affymetrix microarray	Transcriptomic	FGFR 3 /signal transducer and activator of transcription (STAT) pathway and the CSF1R/STAT pathway	↑ *FGF9*<br><br>↓ *ITGB3*, *CSF1*, *MMP19*
**4.**	Krieg et al., 2012 [19]	Decidual	Illumina microarray	Transcriptomic	GO analysis – distinct biological function: immune response (23%), cell signaling (18%) and cell invasion (17.1%)<br><br>pathway analysis revealed consistent changes in both the interleukin 1 (IL-1) and IL-8 pathways	↑ genes in the IL-8 pathway<br><br>↓ genes in the IL-1 pathway
**5.**	Lyu et al., 2013 [19]	Chorionic villi	Microarray	Transcriptomic	↓ mitochondrial function including glutathione<br>↓ oxidative phosphorylation pathway	↓ *ATP6V1F*, *NDUFB1*, *UQCRB*, *ATP5G1*, *ATP5G3*
**6.**	Kosova et al., 2015 [20]	Endometrial biopsy	Histology<br>RNA seq (Illumina Human HT-12)	Transcriptomic	↓ ↑ dysregulation in genes involved in immune response and signalling pathway	-

**7.**	Sõber et al., 2016 [21]	Chorionic villi	RNA-seq (Illumina HiSeq 2000 platform	Transcriptomic	↓ transcript levels of histones, regulatory RNAs and genes involved in telomere, spliceosome, ribosomal, mitochondrial and intra-cellular signalling functions	↑ *ATF4*, *C3*, *PHLDA2*, *GPX4*, *ICAM1*, *SLC16A2*<br><br>↓ *HIST1H1B*, *HIST1H4A*
	**Author**	**Sampled used**	**Analysis method**	**Assay**	**Summary of the findings**	
					**(Main findings)<br>Dysregulated function / pathways in REPL**	**Genes / protein associated with dysregulation**
**8.**	Xin et al., 2016 [22]	Placental villi	mass spectrometry (LC–MS/MS)	Proteomic	↓ Focal adhesion pathway<br>↓ Ribosome pathway	↑ Desmin, Lamin A/C, MMP-9, and histone<br><br>↓ Lamin C/ Lamin A ratio
**9.**	Qiao et al., 2016 [23]	Peripheral blood<br>Chorionic villi	RNA seq (Illumina HiSeq 2000 platform)	Genomic	Complement and coagulation cascade pathway<br>Ciliary motility disorder	↓ ↑ *DYNC2H 1*, *ALOX 15*
**10.**	Huang et al., 2017 [9]	Endometrial biopsy	RNA seq (Illumina HT-12)	Transcriptomic	↓ Complementary and coagulation cascades pathway	↓ *C3*, *C4*, *C4BP*, *DAF*, *DF* and *SERPING1*
**11.**	Altmäe et al., 2017 [24]	Meta-signature genes analysis	Robust rank aggregation analysis	Transcriptomic	importance of immune responses, the complement cascade pathway and the involvement of exosomes	↑ *PAEP*, *SPP1*, *GPX3*, *MAOA* and *GADD45A*<br><br>↓ *SFRP4*, *EDN3*, *OLFM1*, *CRABP2* and *MMP7*.
**12.**	Bahia et al., 2020 [25]	Data extracted from multiple database	Regulatory co-expression network	Transcriptomic DEGs and DEMs	DEMs are highly enriched in TGF-β signaling pathway,Fatty acid metabolism and TNF signaling pathway.	-
**13.**	Li et al., 2020 [26]	Normalized dataset	WGCNA analysis	Transcriptomic	MEred module - cellular defense response and natural killer (NK) cell-mediated cytotoxicity<br>MEbrown module - cell adhesion molecule production, regulation of cellular response to growth factor stimulus, epithelial cell proliferation, and transforming growth factor-β (TGF-β) signaling pathway	*DOCK2*, *TRMT44* and *ERVMER34-1*
**14.**	Lucas et al., 2020 [27]	Endometrial biopsy	RNA-seq	Transcriptomic	-	*SCARA5* and *DIO2*
**15.**	Wang et al., 2020 [28]	Peripheral blood	RNA-seq	Transcriptomic	Cytotoxic properties of CD8T effector, NK, and MAIT cells in peripheral blood indicated	

		Decidua tissue			apparently enhanced immune inflammatory status, and the subpopulation proportions and ligand-receptor interactions of the decidual leukocyte subsets demonstrated preferential immune activation in RSA patients	-

	Author	Sampled used	Analysis method	Assay	Summary of the findings	
					(Main findings)<br>Dysregulated function / pathways in REPL	Genes / protein associated with dysregulation
**16.**	Li et al., 2021 [29]	Decidual tissues	RNA-seq	Transcriptomic	↑ type I interferon signaling pathway<br>↑ TNF signaling pathway	↑ *MX1*, *IFI27*, *ISG15*, and *TNFRSF21*
**17.**	Gu at al., 2021 [30]	Peripheral blood	RNA seq	Transcriptomic	Abnormal expression of immunoregulatory specific gene's function involved in T-cell activation and differentiation.	*TLR2*, *CXCL8*, *IFNG*, *IL2RA*, and *ITGAX*
**18.**	Pearson-Farr et al., 2021 [31]	Endometrial biopsy	RNA seq	Transcriptomic	Dysregulated BP include epithelial cell migration and regulation of secretion by the cell	-
**19.**	Yin et al., 2021 [32]	Decidual tissue	mass spectrometry (LC–MS/MS)	Proteomic	↑ Oxidative phosphorylation	NDUFB3, COX-2

↑ upregulated, ↓ downregulated, ↓ ↑ dysregulated/deregulated

1. Tapia, A.; Gangi, L.M.; Zegers-Hochschild, F.; Balmaceda, J.; Pommer, R.; Trejo, L.; Pacheco, I.M.; Salvatierra, A.M.; Henriquez, S.; Quezada, M.; et al. Differences in the Endometrial Transcript Profile during the Receptive Period between Women Who Were Refractory to Implantation and Those Who Achieved Pregnancy. *Human Reproduction* **2007**, *23*, 340–351, doi:10.1093/humrep/dem319.
2. Koler, M.; Achache, H.; Tsafrir, A.; Smith, Y.; Revel, A.; Reich, R. Disrupted Gene Pattern in Patients with Repeated in Vitro Fertilization (IVF) Failure. *Human Reproduction* **2009**, *24*, 2541–2548, doi:10.1093/humrep/dep193.
3. Lédée, N.; Munaut, C.; Aubert, J.; Sérazin, V.; Rahmati, M.; Chaouat, G.; Sandra, O.; Foidart, J.M. Specific and Extensive Endometrial Deregulation Is Present before Conception in IVF/ICSI Repeated Implantation Failures (IF) or Recurrent Miscarriages: Preconceptional Endometrial Environment Affects Implantation in Humans. *J. Pathol.* **2011**, *225*, 554–564, doi:10.1002/path.2948.
4. Revel, A.; Achache, H.; Stevens, J.; Smith, Y.; Reich, R. MicroRNAs Are Associated with Human Embryo Implantation Defects. *Human Reproduction* **2011**, *26*, 2830–2840, doi:10.1093/humrep/der255.
5. Manohar, M.; Khan, H.; Sirohi, V.K.; Das, V.; Agarwal, A.; Pandey, A.; Siddiqui, W.A.; Dwivedi, A. Alteration in Endometrial Proteins during Early- and Mid-Secretory Phases of the Cycle in Women with Unexplained Infertility. *PLoS ONE* **2014**, *9*, e111687, doi:10.1371/journal.pone.0111687.
6. Koot, Y.E.M.; van Hooff, S.R.; Boomsma, C.M.; van Leenen, D.; Groot Koerkamp, M.J.A.; Goddijn, M.; Eijkemans, M.J.C.; Fauser, B.C.J.M.; Holstege, F.C.P.; Macklon, N.S. An Endometrial Gene Expression Signature Accurately Predicts Recurrent Implantation Failure after IVF. *Sci Rep* **2016**, *6*, 19411, doi:10.1038/srep19411.
7. Choi, Y.; Kim, H.-R.; Lim, E.J.; Park, M.; Yoon, J.A.; Kim, Y.S.; Kim, E.-K.; Shin, J.-E.; Kim, J.H.; Kwon, H.; et al. Integrative Analyses of Uterine Transcriptome and MicroRNAome Reveal Compromised LIF-STAT3 Signaling and Progesterone Response in the Endometrium of Patients with Recurrent/Repeated Implantation Failure (RIF). *PLoS ONE* **2016**, *11*, e0157696, doi:10.1371/journal.pone.0157696.
8. Shi, C.; Shen, H.; Fan, L.-J.; Guan, J.; Zheng, X.-B.; Chen, X.; Liang, R.; Zhang, X.-W.; Cui, Q.-H.; Sun, K.-K.; et al. Endometrial MicroRNA Signature during the Window of Implantation Changed in Patients with Repeated Implantation Failure. *Chinese Medical Journal* **2017**, *130*, 566–573, doi:10.4103/0366-6999.200550.
9. Huang, J.; Qin, H.; Yang, Y.; Chen, X.; Zhang, J.; Laird, S.; Wang, C.C.; Chan, T.F.; Li, T.C. A Comparison of Transcriptomic Profiles in Endometrium during Window of Implantation between Women with Unexplained Recurrent Implantation Failure and Recurrent Miscarriage. *Reproduction* **2017**, *153*, 749–758, doi:10.1530/REP-16-0574.
10. Maekawa, R.; Taketani, T.; Mihara, Y.; Sato, S.; Okada, M.; Tamura, I.; Jozaki, K.; Kajimura, T.; Asada, H.; Tamura, H.; et al. Thin Endometrium Transcriptome Analysis Reveals a Potential Mechanism of Implantation Failure. *Reprod Med Biol* **2017**, *16*, 206–227, doi:10.1002/rmb2.12030.
11. Demiral, I.; Bastu, E.; Gunel, T.; Sezerman, U.; Gumusoglu, E.; Ulgen, E.; Hosseini, M.K.; Buyru, F.; Yeh, J. Endometrial Gene Expression in Patients with Recurrent Implantation Failure. *Fertility and Sterility* **2017**, *108*, e365, doi:10.1016/j.fertnstert.2017.07.1068.
12. Bastu, E.; Demiral, I.; Gunel, T.; Ulgen, E.; Gumusoglu, E.; Hosseini, M.K.; Sezerman, U.; Buyru, F.; Yeh, J. Potential Marker Pathways in the Endometrium That May Cause

Recurrent Implantation Failure. *Reprod Sci* **2019**, *26*, 879–890, doi:10.1177/1933719118792104.
13. Shi, C.; Han, H.J.; Fan, L.J.; Guan, J.; Zheng, X.B.; Chen, X.; Liang, R.; Zhang, X.W.; Sun, K.K.; Cui, Q.H.; et al. Diverse Endometrial MRNA Signatures during the Window of Implantation in Patients with Repeated Implantation Failure. *Human Fertility* **2018**, *21*, 183–194, doi:10.1080/14647273.2017.1324180.
14. Wang, F.; Liu, Y. Identification of Key Genes, Regulatory Factors, and Drug Target Genes of Recurrent Implantation Failure (RIF). *Gynecological Endocrinology* **2020**, *36*, 448–455, doi:10.1080/09513590.2019.1680622.
15. Gao, M.; Kang, Y.; Li, B.; Fan, S.; Zhang, X. *Bioinformatics of Potential Biomarkers in Patients with Repeated Implantation Failure*; Sexual and Reproductive Health, 2020;
16. Wang, C.; Feng, Y.; Zhou, W.-J.; Cheng, Z.-J.; Jiang, M.-Y.; Zhou, Y.; Fei, X.-Y. Screening and Identification of Endometrial Proteins as Novel Potential Biomarkers for Repeated Implantation Failure. *PeerJ* **2021**, *9*, e11009, doi:10.7717/peerj.11009.
17. Lee, J.; Oh, J.; Choi, E.; Park, I.; Han, C.; Kim, D.H.; Choi, B.-C.; Kim, J.-W.; Cho, C. Differentially Expressed Genes Implicated in Unexplained Recurrent Spontaneous Abortion. *Int J Biochem Cell Biol* **2007**, *39*, 2265–2277, doi:10.1016/j.biocel.2007.06.012.
18. Othman, R.; Omar, M.H.; Shan, L.P.; Shafiee, M.N.; Jamal, R.; Mokhtar, N.M. Microarray Profiling of Secretory-Phase Endometrium from Patients with Recurrent Miscarriage. *Reprod Biol* **2012**, *12*, 183–199, doi:10.1016/s1642-431x(12)60085-0.
19. Lyu, S.W.; Song, H.; Yoon, J.A.; Chin, M.-U.; Sung, S.R.; Kim, Y.S.; Lee, W.S.; Yoon, T.K.; Cha, D.H.; Shim, S.H. Transcriptional Profiling with a Pathway-Oriented Analysis in the Placental Villi of Unexplained Miscarriage. *Placenta* **2013**, *34*, 133–140, doi:10.1016/j.placenta.2012.12.003.
20. Kosova, G.; Stephenson, M.D.; Lynch, V.J.; Ober, C. Evolutionary Forward Genomics Reveals Novel Insights into the Genes and Pathways Dysregulated in Recurrent Early Pregnancy Loss. *Hum Reprod* **2015**, *30*, 519–529, doi:10.1093/humrep/deu355.
21. Sõber, S.; Rull, K.; Reiman, M.; Ilisson, P.; Mattila, P.; Laan, M. RNA Sequencing of Chorionic Villi from Recurrent Pregnancy Loss Patients Reveals Impaired Function of Basic Nuclear and Cellular Machinery. *Sci Rep* **2016**, *6*, 38439, doi:10.1038/srep38439.
22. Xin, L.; Xu, B.; Ma, L.; Hou, Q.; Ye, M.; Meng, S.; Ding, X.; Ge, W. Proteomics Study Reveals That the Dysregulation of Focal Adhesion and Ribosome Contribute to Early Pregnancy Loss. *Prot. Clin. Appl.* **2016**, *10*, 554–563, doi:10.1002/prca.201500136.
23. Qiao, Y.; Wen, J.; Tang, F.; Martell, S.; Shomer, N.; Leung, P.C.K.; Stephenson, M.D.; Rajcan-Separovic, E. Whole Exome Sequencing in Recurrent Early Pregnancy Loss. *Mol Hum Reprod* **2016**, *22*, 364–372, doi:10.1093/molehr/gaw008.
24. Altmäe, S.; Koel, M.; Võsa, U.; Adler, P.; Suhorutšenko, M.; Laisk-Podar, T.; Kukushkina, V.; Saare, M.; Velthut-Meikas, A.; Krjutškov, K.; et al. Meta-Signature of Human Endometrial Receptivity: A Meta-Analysis and Validation Study of Transcriptomic Biomarkers. *Sci Rep* **2017**, *7*, 10077, doi:10.1038/s41598-017-10098-3.
25. Bahia, W.; Soltani, I.; Abidi, A.; Haddad, A.; Ferchichi, S.; Menif, S.; Almawi, W.Y. Identification of Genes and MiRNA Associated with Idiopathic Recurrent Pregnancy Loss: An Exploratory Data Mining Study. *BMC Med Genomics* **2020**, *13*, 75, doi:10.1186/s12920-020-00730-z.
26. Li, X.; He, Y.; Hao, C.; Li, X.; Li, X. Weighted Gene Correlation Network Analysis Reveals Novel Regulatory Modules Associated with Recurrent Early Pregnancy Loss. *Bioscience Reports* **2020**, *40*, BSR20193938, doi:10.1042/BSR20193938.

27. Lucas, E.S.; Vrljicak, P.; Muter, J.; Diniz-da-Costa, M.M.; Brighton, P.J.; Kong, C.-S.; Lipecki, J.; Fishwick, K.J.; Odendaal, J.; Ewington, L.J.; et al. Recurrent Pregnancy Loss Is Associated with a Pro-Senescent Decidual Response during the Peri-Implantation Window. *Commun Biol* **2020**, *3*, 37, doi:10.1038/s42003-020-0763-1.
28. Wang, F.; Jia, W.; Fan, M.; Shao, X.; Li, Z.; Liu, Y.; Ma, Y.; Li, Y.-X.; Li, R.; Tu, Q.; et al. Single-Cell Immune Landscape of Human Recurrent Miscarriage. *Genomics Proteomics Bioinformatics* **2021**, *19*, 208–222, doi:10.1016/j.gpb.2020.11.002.
29. Li, Y.; Wang, R.; Wang, M.; Huang, W.; Liu, C.; Fang, Z.; Liao, S.; Jin, L. RNA Sequencing of Decidua Reveals Differentially Expressed Genes in Recurrent Pregnancy Loss. *Reprod Sci* **2021**, *28*, 2261–2269, doi:10.1007/s43032-021-00482-w.
30. Gu, H.; Li, L.; Du, M.; Xu, H.; Gao, M.; Liu, X.; Wei, X.; Zhong, X. Key Gene and Functional Pathways Identified in Unexplained Recurrent Spontaneous Abortion Using Targeted RNA Sequencing and Clinical Analysis. *Front. Immunol.* **2021**, *12*, 717832, doi:10.3389/fimmu.2021.717832.
31. Pearson-Farr, J.; Lewis, R.; Cleal, J.; Cheong, Y. P–555 Recurrent Pregnancy Loss Is Associated with Changes in the Pre-Pregnant Endometrial Gland Transcriptome. *Human Reproduction* **2021**, *36*, deab130.554, doi:10.1093/humrep/deab130.554.
32. Yin, X.-J.; Hong, W.; Tian, F.-J.; Li, X.-C. Proteomic Analysis of Decidua in Patients with Recurrent Pregnancy Loss (RPL) Reveals Mitochondrial Oxidative Stress Dysfunction. *Clin Proteom* **2021**, *18*, 9, doi:10.1186/s12014-021-09312-2.
